# Modal approximation for plasmonic resonators in the time domain: the scalar case

**DOI:** 10.1007/s42985-021-00098-4

**Published:** 2021-06-10

**Authors:** Lorenzo Baldassari, Pierre Millien, Alice L. Vanel

**Affiliations:** 1 Department of Mathematics, ETH Zürich, Rämistrasse 101, 8092 Zurich, Switzerland; 2 Institut Langevin, ESPCI Paris, PSL University, CNRS, 1 Rue Jussieu, 75005 Paris, France

**Keywords:** Plasmonic resonance, Time-domain modal expansion, Subwavelength resonators, Quasi-normal modes, 35R30, 35C20

## Abstract

We study the electromagnetic field scattered by a metallic nanoparticle with dispersive material parameters in a resonant regime. We consider the particle placed in a homogeneous medium in a low-frequency regime. We define modes for the non-Hermitian problem as perturbations of electro-static modes, and obtain a modal approximation of the scattered field in the frequency domain. The poles of the expansion correspond to the eigenvalues of a singular boundary integral operator and are shown to lie in a bounded region near the origin of the lower-half complex plane. Finally, we show that this modal representation gives a very good approximation of the field in the time domain. We present numerical simulations in two dimensions to corroborate our results.

## Introduction

### Context

When describing the interaction of light with a resonating particle, summing the natural resonant modes of the system is an intuitive and attractive approach. The modes are easily computed as they are eigenmode solutions to a source-free problem. They are intrinsic quantities of the system and give insights to understand the underlying physics. Once they are calculated, the response of the system to any given excitation can be computed at a low computational cost. A bounded, lossless system is Hermitian and admits a basis of orthonormal eigenmodes associated to real eigenvalues. But for a system that exhibits loss (by absorption or radiation), the classical spectral theorem cannot be used to diagonalise the non-Hermitian operator and the eigenvalues become complex [28, 33, 45].

Several authors have obtained modal expansions for non-Hermitian systems [15, 22, 25, 29, 34, 37, 38, 43, 49]. Their use in nanophotonics is quite recent and is studied by many research groups in the physics community (see the review paper [28] and references therein). Nevertheless, a number of theoretical and numerical issues arise [14, 17]. Modes of non-Hermitian systems are not orthogonal, using classical inner products. In order to satisfy the outgoing boundary conditions, these generalised modes have complex frequencies with negative imaginary parts and, if they decay exponentially in time as $t \rightarrow \infty$, they grow far away from the resonating systems. This is known in the literature as Lamb’s exponential catastrophe [40]. Recently, frameworks for the computation and normalisation of these generalised modes have been established in different settings [21, 26, 41–43, 47].

### Scope of the paper

In this paper we consider the scattering of a scalar wave by an obstacle with dispersive parameters (described by a *Drude–Lorentz model*). This is a good model for the scattering of light by a dispersive obstacle in the *transverse magnetic polarisation* (see [32, remark 2.1]). We work in a *low-frequency regime* corresponding to relevant physical applications, such as the scattering of light in the visible/infrared domain by a metallic nanoparticle whose characteristic size is a few tens of nanometers.

The goal of this paper is to obtain an *approximation* of the *low-frequency part* of the scattered field by a dispersive obstacle in the time domain as a *finite sum* of modes oscillating at complex frequencies.

The tools used are singular boundary integral equations and elementary functional analysis. In this paper, we do not deal with the high frequency part of the field that is usually studied with micro-local analysis tools.

### Previous work on plasmonic resonances and layer potentials

It has been shown in [1, 6, 8] that using boundary integral representation and layer potential analysis, one can define the resonant frequencies as solutions of a non-linear eigenvalue problem on the boundary of the particle. In a low-frequency regime, i.e., at frequencies corresponding to wavelengths that are orders of magnitude larger than the particle’s size, asymptotic analysis techniques, as in [6], yield a hierarchy of boundary integral equations. The asymptotic small parameter is $\delta \omega c^{-1}$, where $\delta$ is the size of the particle, $\omega$ the frequency and *c* the velocity. At leading order the well-known Neumann–Poincaré operator appears [48]. Using the Plemelj symmetrisation principle and the spectral theory of compact self-adjoint operators, the latter can be diagonalised in the appropriate functional spaces [24, 35], which allows the scattered field to be decomposed in a basis of orthogonal modes in the static case [10]. The properties of the eigenvalues of the Neumann–Poincaré operator have been extensively studied in the literature, see the review paper [13] and references therein. For a smooth enough boundary, say $C^{1,\alpha }$ for some $\alpha >0$, the operator is compact and its eigenvalues are real numbers converging to zero. The eigenvalues of the Neumann–Poincaré operator in the two- and three-dimensional cases are intrinsically different. In two dimensions, the spectrum is symmetric with respect to the origin (except for the eigenvalue 1/2), so there are as many positive eigenvalues as negative. The decay rate of the eigenvalues depends strongly on the regularity of the boundary. For an analytic boundary, the eigenvalues have an exponential decay rate [11]. In three dimensions, very few surfaces are known to have negative eigenvalues [23]. For a strictly convex $C^\infty$ domain, there are infinitely many positive eigenvalues and a finite number of negatives ones [12]. The eigenvalues rate of decay is much slower than in two dimensions: $\lambda _j={\mathcal {O}}(j^{-1/2})$ as $j\rightarrow \infty$ [31].

### Contributions and organisation of the paper

We begin by describing the problem geometry and we formulate the governing equations in Sect. 2. We introduce the layer potential and boundary integral formulation and recall the modal decomposition of the static ($\omega =0$) solution. In Sect. 3, we prove that in three dimensions, for a strictly convex particle, the modal expansion can be truncated due to the super-polynomial decay of the expansion’s coefficients. With a perturbation argument, we deduce from the static ($\omega = 0$) result a modal approximation in the dynamic case (for a small non-zero frequency). The perturbation analysis yields size dependent dynamic complex resonant frequencies. We show that all the resonant frequencies have a negative imaginary part and lie in a bounded region near the origin. Finally, in Sect. 5, using only elementary complex analysis techniques, we give an approximation for the low-frequency part of the scattered field in the time domain as a finite sum of modes oscillating at complex resonant frequencies. We also show with a simple causality argument that the *exponential catastrophe* is not problematic in practice. In Sect. 6, we implement this expansion in the two-dimensional setting and illustrate the validity of our approach with numerical simulations.

## Problem geometry and formulation

### Problem setting

We are interested in the scattering problem of an incident wave illuminating a plasmonic nanoparticle in ${\mathbb {R}}^d$, $d=2,3$. The homogeneous medium is characterised by electric permittivity $\varepsilon _m$ and magnetic permeability $\mu _m$. Let *D* be a smooth bounded domain in ${\mathbb {R}}^d$, of class $C^\infty$, characterised by electric permittivity $\varepsilon _c$. We assume the particle to be non-magnetic, i.e., $\mu _c = \mu _m$. Let $D=z+\delta B$ where *B* is the reference domain and contains the origin, and *D* is located at $z\in {\mathbb {R}}^d$ and has a characteristic size $\delta \ll 1$. We define the wavenumbers $k_c=\omega \sqrt{\varepsilon _c\mu _c}$ and $k_m=\omega \sqrt{\varepsilon _m\mu _m}$. Let $\varepsilon =\varepsilon _c \chi (D)+\varepsilon _m \chi ({\mathbb {R}}^d {\setminus } {\bar{D}})$, where $\chi$ denotes the characteristic function. We denote by $c_0$ the speed of light in vacuum $c_0=1/\sqrt{\varepsilon _0\mu _0}$ and by *c* the speed of light in the medium $c=1/\sqrt{\varepsilon _m\mu _m}$.

Hereafter we use the Drude model [36] to express the electric permittivity of the particle:

1 $$\begin{aligned} \varepsilon _c(\omega )=\varepsilon _0\left( 1-\frac{\omega _p^2}{\omega ^2+i\omega \mathrm {T}^{-1}}\right) , \end{aligned}$$

where the positive constants $\omega _p$ and $\mathrm {T}^{-1}$ are the plasma frequency and the collision frequency or damping factor, respectively.

#### Condition 1

In two dimensions, we assume the domain *D* to be an algebraic domain of class ${\mathcal {Q}}$, i.e., a quadrature domain. An algebraic domain is a domain enclosed by a real algebraic curve, namely the zero level set of a bivariate polynomial. A quadrature domain is the conformal image of the unit disc by a rational function.

#### Remark 1

Algebraic domains are dense among all planar domains, so every smooth curve can be described as a sequence of algebraic curves [7].

#### Condition 2

In three dimensions, we assume the domain *D* to be strictly convex: for any two points in *D*, the line segment joining them is contained in $D {\setminus } \partial D$.

Throughout the rest of the paper, *D* is assumed to satisfy conditions 1 or 2.

### Helmholtz equation for a subwavelength resonator

Given an incident wave $u^\text {in}$ solution to the Helmholtz equation, the scattering problem in the frequency domain can be modelled by

2 $$\begin{aligned} \nabla \cdot \frac{1}{\varepsilon (x)} \nabla u(x)+\omega ^2\mu _m u(x) = 0, \qquad x \in {\mathbb {R}}^d, \end{aligned}$$

subject to the Sommerfeld radiation condition

$$\begin{aligned} \left| \frac{\partial (u-u^\text {in})}{\partial |x|}-ik_m(u-u^\text {in})\right| ={\mathcal {O}}\left( |x|^{-(d+1)/2}\right) , \qquad \text{ as } |x|\rightarrow \infty , \end{aligned}$$

uniformly in *x*/|*x*|, for $\mathfrak {R}{k_m}>0$. The transmission conditions are given by

$$\begin{aligned} {\left\{ \begin{array}{ll} \left. u(x)\right| _+ = \left. u(x)\right| _-, &{} x \in \partial D,\\ \left. \frac{1}{\varepsilon _m}\frac{\partial u(x)}{\partial \nu }\right| _+ = \left. \frac{1}{\varepsilon _c} \frac{\partial u(x)}{\partial \nu }\right| _-,&{} x \in \partial D. \end{array}\right. } \end{aligned}$$

Here, $\partial \cdot / \partial \nu$ denotes the normal derivative on $\partial D$, and the $+$ and − subscripts indicate the limits from outside and inside *D*, respectively.

#### Definition 1

We denote the contrast $\lambda$ by

$$\begin{aligned} \lambda (\omega )=\frac{\varepsilon _m+\varepsilon _c}{2(\varepsilon _m-\varepsilon _c)}. \end{aligned}$$

#### Definition 2

(*Resonant frequency, mode*) We say $\omega$ is a *resonant frequency* if there is a non-trivial solution to Eq. (2) with $u^\text {in}=0$. We call the solution a mode. A *subwavelength resonance* occurs when a resonant frequency $\omega$ satisfies $\omega \delta c^{-1} <1$.

#### Remark 2

Although the Helmholtz equation does not model light matter interaction in three dimensions, the spectral properties of the Maxwell volume integral operator are closely linked to those of the Neumann–Poincaré operator [5, 19]. Therefore it is insightful to study the three-dimensional case. Moreover, the three-dimensional Helmholtz equation models acoustic waves and after adapting the physical parameters one could use this setting to study bubbles.

### Layer potential formulation

Let $H^{1/2}(\partial D)$ be the usual Sobolev space and let $H^{-1/2}(\partial D)$ be its dual space with respect to the duality pairing $\left\langle \cdot ,\cdot \right\rangle _{\frac{1}{2},-\frac{1}{2}}$. The field *u* can be represented using the single-layer potentials ${\mathcal {S}}^{k_c}_D$ and ${\mathcal {S}}^{k_m}_D$, introduced in Definition 8, as follows:

3 $$\begin{aligned} u(x)={\left\{ \begin{array}{ll} {\mathcal {S}}^{k_c}_D[\varPhi ](x), &{} x\in D,\\ u^\text {in}(x)+{\mathcal {S}}^{k_m}_D[\varPsi ](x), &{} x\in {\mathbb {R}}^d {\setminus } {\overline{D}}, \end{array}\right. } \end{aligned}$$

where the pair $(\varPhi , \varPsi ) \in H^{-\frac{1}{2}}(\partial D)\times H^{-\frac{1}{2}}(\partial D)$ is the unique solution to

4 $$\begin{aligned} {\left\{ \begin{array}{ll} {\mathcal {S}}^{k_m}_D[\varPsi ](x)-{\mathcal {S}}^{k_c}_D[\varPhi ](x)=F_1,&{} x\in \partial D,\\ \frac{1}{\varepsilon _m}\left( \frac{1}{2}I+{\mathcal {K}}^{k_m,*}_D\right) [\varPsi ](x)+\frac{1}{\varepsilon _c}\left( \frac{1}{2}I-{\mathcal {K}}^{k_c,*}_D\right) [\varPhi ](x)=F_2, &{} x\in \partial D, \end{array}\right. } \end{aligned}$$

and

$$\begin{aligned} F_1=-u^\text {in}(x), \qquad F_2=-\frac{1}{\varepsilon _m}\frac{\partial u^\text {in}(x)}{\partial \nu }, \qquad x \in \partial D, \end{aligned}$$

where ${\mathcal {K}}^{k_m,*}_D$ is the Neumann–Poincaré operator introduced in Definition 8. The trace relations for the single-layer potential are given in Lemma 6.

### Scaling and small-volume approximation

The goal of this section is to establish an equivalent formulation for (4) in the form ${\mathcal {A}}^{\omega \delta c^{-1}}_B[\varPsi ]= F$ (Proposition 1), in order to write an asymptotic expansion of the operator ${\mathcal {A}}^{\omega \delta c^{-1}}_B$ (Lemma 1) and a spectral decomposition for the limiting operator ${\mathcal {A}}^{0}_B$ (Proposition 2). The scaling is new in this context, but the asymptotic expansion and the spectral decomposition were first obtained in [6]. We recall them here for the sake of completeness. The proofs are quite lengthy and technical, so they are included in the appendix.

Recall that *z* is the centre of the resonator and $\delta$ its radius. We introduce the scaling $x=z+\delta X$. For each function $\varXi$ defined on $\partial D$, we define a corresponding function on $\partial B$ by ${\widetilde{\varXi }}(X) := \varXi (z+\delta X)$, $X \in \partial B$. The scaling properties of the integral operators are given in Appendix B. The solution ${\widetilde{u}}$ becomes

5 $$\begin{aligned} {\widetilde{u}}(X)={\left\{ \begin{array}{ll} \delta {\mathcal {S}}^{k_c\delta }_B[{\widetilde{\varPhi }}](X), &{} X\in B,\\ u^\text {in}(z+\delta X)+\delta {\mathcal {S}}^{k_m\delta }_B[{\widetilde{\varPsi }}](X), &{} X\in {\mathbb {R}}^d {\setminus } {\overline{B}}, \end{array}\right. } \end{aligned}$$

where the single-layer potential ${\mathcal {S}}^{k\delta }_B$ and Neumann–Poincaré operator ${\mathcal {K}}^{k\delta ,*}_B$ are defined by the fundamental solution $\varGamma ^{k\delta }$. The density pair $({\widetilde{\varPhi }},{\widetilde{\varPsi }}) \in H^{-\frac{1}{2}}(\partial B)\times H^{-\frac{1}{2}}(\partial B)$ is the unique solution to

$$\begin{aligned} {\left\{ \begin{array}{ll} {\mathcal {S}}^{k_m\delta }_B[{\widetilde{\varPsi }}](X)-{\mathcal {S}}^{k_c\delta }_B[{\widetilde{\varPhi }}](X)=\frac{1}{\delta } {\widetilde{F}}_1, &{} X\in \partial B,\\ \frac{1}{\varepsilon _m}\left( \frac{1}{2}I+{\mathcal {K}}^{k_m\delta ,*}_B\right) [{\widetilde{\varPsi }}](X)+\frac{1}{\varepsilon _c}\left( \frac{1}{2}I-{\mathcal {K}}^{k_c\delta ,*}_B\right) [{\widetilde{\varPhi }}](X)={\widetilde{F}}_2,&{} X\in \partial B, \end{array}\right. } \end{aligned}$$

and

$$\begin{aligned} {\widetilde{F}}_1=-u^\text {in}(z+\delta X), \qquad {\widetilde{F}}_2=-\frac{1}{\delta \varepsilon _m}\frac{\partial u^\text {in}(z+\delta X)}{\partial \nu _X}, \qquad X \in \partial B. \end{aligned}$$

Since ${\mathcal {S}}_{B}^{k_c\delta }:H^{-1/2} (\partial B)\rightarrow H^{1/2}(\partial B)$ is invertible for $k_c\delta$ small enough (see Lemmas 7 and 10 ), the following proposition holds.

#### Proposition 1

For $d=2,3$, the following equation holds for ${\widetilde{\varPsi }}$:

6 $$\begin{aligned} {\mathcal {A}}_B^{\omega \delta c^{-1}}[{\widetilde{\varPsi }}]={\widetilde{F}}, \end{aligned}$$

where

7 $$\begin{aligned} {\mathcal {A}}_B^{\omega \delta c^{-1}}= & {} \frac{1}{\varepsilon _m}\left( \frac{1}{2}I+{\mathcal {K}}^{k_m\delta ,*}_B\right) +\frac{1}{\varepsilon _c}\left( \frac{1}{2}I-{\mathcal {K}}^{k_c\delta ,*}_B\right) \left( {\mathcal {S}}^{k_c\delta }_B\right) ^{-1}{\mathcal {S}}^{k_m\delta }_B, \nonumber \\ {\widetilde{F}}= & {} {\widetilde{F}}_2+\frac{1}{\delta \varepsilon _c}\left( \frac{1}{2}I-{\mathcal {K}}^{k_c\delta ,*}_B\right) \left( {\mathcal {S}}^{k_c\delta }_B\right) ^{-1}[{\widetilde{F}}_1]. \end{aligned}$$

#### Lemma 1

(Small-volume expansion) As $\omega \delta c^{-1}\rightarrow 0$, ${\mathcal {A}}_B^{\omega \delta c^{-1}}$ admits the following asymptotic expansion:

8 $$\begin{aligned} {\mathcal {A}}_B^{\omega \delta c^{-1}}= {\left\{ \begin{array}{ll} {\mathcal {A}}_B^{0}+\left( \omega \delta c^{-1}\right) ^2\log {\left( \omega \delta c^{-1}\right) }{\mathcal {A}}_{B,1}+{\mathcal {O}}\left( \left( \omega \delta c^{-1}\right) ^2\right) , &{} d=2, \\ {\mathcal {A}}_B^{0}+\left( \omega \delta c^{-1}\right) ^2{\mathcal {A}}_{B,2}+{\mathcal {O}}\left( \left( \omega \delta c^{-1}\right) ^3\right) , &{} d=3, \end{array}\right. } \end{aligned}$$

where

9 $$\begin{aligned} {\mathcal {A}}_B^{0}= & {} \left( \frac{1}{2\varepsilon _c}+\frac{1}{2\varepsilon _m}\right) I-\left( \frac{1}{\varepsilon _c}-\frac{1}{\varepsilon _m}\right) {\mathcal {K}}^*_B, \nonumber \\ {\mathcal {A}}_{B,1}= & {} \frac{1}{\varepsilon _m} {\mathcal {K}}^{(1)}_{B,1}(I -{\mathcal {P}}_{{\mathcal {H}}^*_0}) + \left( \frac{1}{2}I - {\mathcal {K}}^*_B\right) \widetilde{{\mathcal {S}}}_B^{-1} {\mathcal {S}}_{B,1}^{(1)} \left( \frac{1}{\varepsilon _c}I - \frac{1}{\varepsilon _m } {\mathcal {P}}_{{\mathcal {H}}^*_0} \right) , \end{aligned}$$

and

$$\begin{aligned} {\mathcal {A}}_{B,2}=\frac{\varepsilon _m-\varepsilon _c}{\varepsilon _m\varepsilon _c}\left( \frac{1}{2}I-{\mathcal {K}}^*_B\right) {\mathcal {S}}_B^{-1}{\mathcal {S}}_{B,2}, \end{aligned}$$

where the operators ${\mathcal {P}}_{{\mathcal {H}}^*_0}, \; \widetilde{{\mathcal {S}}}_B, \; {\mathcal {S}}_{B,1}^{(1)}, \; {\mathcal {S}}_{B,2}$ and ${\mathcal {K}}^{(1)}_{B,1}$ are defined in Appendix C.1.

#### Proof

See Appendix C.2. $$\square $$

The operator ${\mathcal {A}}_B^{\omega \delta c^{-1}}$ is not self-adjoint in $L^2$ so it can not be diagonalised directly to solve (6). However, in the static regime, the operator ${\mathcal {A}}_B^0$ can be expressed simply with ${\mathcal {K}}^*_B$, which can be symmetrised in the Hilbert space ${\mathcal {H}}^*(\partial B)$ (see Appendix A.2).

#### Lemma 2

(Spectral decomposition of ${\mathcal {K}}_B^*$) ${\mathcal {K}}^*_B$ is self-adjoint with respect to the inner product $\left\langle \cdot ,\cdot \right\rangle _{{\mathcal {H}}^*(\partial B)}$. Moreover, it is compact, so its spectrum is discrete. The spectral theorem yields the decomposition

$$\begin{aligned} {\mathcal {K}}^*_B=\sum _{j=0}^{+\infty } \lambda _j\left\langle \cdot ,{\widetilde{\phi }}_j\right\rangle _{{\mathcal {H}}^*(\partial B)}{\widetilde{\phi }}_j, \end{aligned}$$

where $\{\lambda _j\}_{j\in {\mathbb {N}}}$ are the eigenvalues of ${\mathcal {K}}^*_B$ and $\{{\widetilde{\phi }}_j\}_{j\in {\mathbb {N}}}$ their associated normalised eigenvectors.

#### Proposition 2

(Spectral decomposition of ${\mathcal {A}}_B^0$) The operator ${\mathcal {A}}_B^0$ has the spectral decomposition

$$\begin{aligned} {\mathcal {A}}_B^0=\sum _{j=0}^{+\infty } \tau _j\left\langle \cdot ,{\widetilde{\phi }}_j\right\rangle _{{\mathcal {H}}^*(\partial B)}{\widetilde{\phi }}_j, \end{aligned}$$

where $(\lambda _j,{\widetilde{\phi }}_j)_{j\in {\mathbb {N}}}$ are the eigenvalues and normalised eigenfunctions of ${\mathcal {K}}_B^*$ in ${\mathcal {H}}^*(\partial B)$ and

$$\begin{aligned} \tau _j=\left( \frac{1}{\varepsilon _c}-\frac{1}{\varepsilon _m}\right) \left( \lambda (\omega )-\lambda _j\right) . \end{aligned}$$

#### Proof

Direct consequence of Lemma 2 and (9). $\square$

#### Corollary 1

The spectral approximation of the static ($\omega =0$) solution is given by

$$\begin{aligned} {\widetilde{u}}(X)-{\widetilde{u}}^\text {in}(X) = \sum _{j=0}^{\infty }\frac{1}{\tau _j} \left\langle {\widetilde{F}},{\widetilde{\phi }}_j\right\rangle _{{\mathcal {H}}^*(\partial B)} \delta {\mathcal {S}}_B[{\widetilde{\phi }}_j](X), \qquad X \in {\mathbb {R}}^d {\setminus } {\overline{B}}, \end{aligned}$$

where ${\widetilde{F}}$ is defined in Proposition 1.

## Modal decomposition of the field

In this section, we want to apply perturbation theory tools to express the solution of (6) in terms of the eigenvectors of ${\mathcal {K}}^*_B$ that appear in the spectral decomposition of the limiting problem in Proposition 2, and to replace $\tau _j$ by a perturbed value $\tau _j(\omega \delta c^{-1})$. Classical perturbation theory will give us a Taylor expansion for $\tau _j(\omega \delta c^{-1})$ in $\omega \delta c^{-1}$ for any $j\in {\mathbb {N}}$ but the remainders and validity range of these expansions will depend on the index *j* of the considered eigenvalue. In order to get a meaningful expansion of the scattered field we need to work with a finite number of modes.

### Modal expansion truncation

In practice, there is no need to consider the whole spectral decomposition of the field. It has been empirically reported that only a few modes actually contribute to the scattered field. The number of modes to consider increases as the source gets closer to the particle. In this section we give a mathematical explanation of this phenomenon : the modes ${\widetilde{\phi }}_j$ are eigenmodes of a pseudo-differential operator of order $-1$, and are oscillating functions. As in classical Fourier analysis, the decay with *j* of the coefficients $\langle {\widetilde{F}},{\widetilde{\phi }}_j\rangle _{{\mathcal {H}}^*(\partial B)}$ will be determined by the regularity of the function ${\widetilde{F}}$ and the number of modes to consider will depend on the spatial variations of ${\widetilde{F}}$ over $\partial B$. In an homogeneous medium the incoming field is smooth and therefore we can expect a fast decay of the coefficients.

#### Remark 3

(On high-order modes) The oscillation rate of high-order modes has been quantified in the case of smooth axisymmetric inclusions [39]. Moreover, it has been also shown in that case that the contribution of high-order modes to the electric field outside the particle decays exponentially as the distance from the inclusion increases. This property has also been shown in [12]. This suggests that not only high-order modes are not really excited by a source bounded away from the particle because of their oscillating properties, but they also do not contribute to the scattered field except in the near field.

#### Remark 4

In this work we do not use the exponential decay of the modes far away from the particle to justify the truncation of the quasi–static modal expansion because the exponential decay has only been shown for the *electro-static modes*, i.e., the quantities ${\mathcal {S}}_D^0[\phi _j]$ as $j \rightarrow \infty$, while our expansions uses *dynamic modes* ${\mathcal {S}}_D^\omega [\phi _j]$. Nevertheless we think that the property still holds for $\omega \not = 0$.

#### The three-dimensional case

Let $H^{S}$ be the standard Sobolev space of order *S*.

##### Proposition 3

For *B*, a strictly convex domain in ${\mathbb {R}}^3$ with $C^\infty$-smooth boundary, and ${\widetilde{F}}\in H^S(\partial B)$ for some $S \in {\mathbb {N}}^*$ we have :

10 $$\begin{aligned} \left\langle {\widetilde{F}}, {\widetilde{\phi }}_j\right\rangle _{{\mathcal {H}}^*(\partial B)}=o(j^{-S/4}) \text {~as~} j\rightarrow +\infty . \end{aligned}$$

The proof relies on a theorem from [12] which itself uses the computation of the principal symbol of the Neumann–Poincaré operator done in [30]:

##### Theorem 1

(From [12], p. 7) For *B*, a strictly convex domain in ${\mathbb {R}}^3$ with $C^\infty$-smooth boundary, ${\mathcal {K}}_B^*$ has a finite number of non-positive eigenvalues. We can modify ${\mathcal {K}}_B^*$ by adding a finite dimensional smoothing operator to have a positive definite elliptic pseudo-differential operator of order − 1, which we denote by $\widetilde{{\mathcal {K}}}_B^*$. For each real number $s\in {\mathbb {R}}$ there exist constants $c_s,C_s\in {\mathbb {R}}^+$ such that

11 $$\begin{aligned} c_s||{\widetilde{\phi }}||_{H^{s-1/2}(\partial B)} \le ||\widetilde{{\mathcal {K}}}_B^*[{\widetilde{\phi }}]||_{H^{s+1/2}(\partial B)}\le C_s||{\widetilde{\phi }}||_{H^{s-1/2}(\partial B)} \end{aligned}$$

for all ${\widetilde{\phi }} \in H^{s-1/2}(\partial B)$. Moreover there exists $j_0\in {\mathbb {N}}$ such that

$$\begin{aligned} \widetilde{{\mathcal {K}}}_B^*[{\widetilde{\phi }}_j]={\mathcal {K}}_B^*[{\widetilde{\phi }}_j] \quad \text {and}\quad \lambda _j>0 \quad \text {for all }\quad j \ge j_0. \end{aligned}$$

##### Corollary 2

The operator ${\mathbf {K}}^*_B: L^2(\partial B) \longrightarrow L^2(\partial B)$ defined by ${\mathbf {K}}^*_B:=\left( -{\mathcal {S}}_B\right) ^{\frac{1}{2}} {\mathcal {K}}_B^* \left( -{\mathcal {S}}_B\right) ^{-\frac{1}{2}}$ is self-adjoint and has the same eigenvalues as ${\mathcal {K}}^*_B$. Its eigenvectors are ${\widetilde{\psi }}_j=\left( -{\mathcal {S}}_B\right) ^{\frac{1}{2}}[{\widetilde{\phi }}_j]$. It can be modified by adding a finite dimensional smoothing operator to have a positive definite elliptic pseudo-differential operator of order $-1$, which we denote by $\widetilde{{\mathbf {K}}}_B^*$. For each real number $s\in {\mathbb {R}}$ there exist constants $c_s,C_s\in {\mathbb {R}}^+$ such that

12 $$\begin{aligned} c_s||{\widetilde{\phi }}||_{H^{s-1/2}(\partial B)} \le ||\widetilde{{\mathbf {K}}}_B^*[{\widetilde{\phi }}]||_{H^{s+1/2}(\partial B)}\le C_s||{\widetilde{\phi }}||_{H^{s-1/2}(\partial B)} \end{aligned}$$

for all ${\widetilde{\phi }} \in H^{s-1/2}(\partial B)$. Moreover there exists $j_0\in {\mathbb {N}}$ such that

$$\begin{aligned} \widetilde{{\mathbf {K}}}_B^*[{\widetilde{\psi }}_j]={\mathbf {K}}_B^*[{\widetilde{\psi }}_j] \quad \text {and}\quad \lambda _j>0 \quad \text {for all }\quad j \ge j_0. \end{aligned}$$

##### Proof

${\mathbf {K}}^*_B$ has the same principal symbol as ${\mathcal {K}}_B^*$ [31, p. 8]. $\square$

We will also need the decay estimate of the eigenvalues of ${\mathcal {K}}_B^*$:

##### Theorem 2

(From [31]) For *B*, a strictly convex domain in ${\mathbb {R}}^3$ with $C^\infty$-smooth boundary the eigenvalues of the Neumann–Poincaré operator satisfy:

$$\begin{aligned} \lambda _j \sim C_B j^{-1/2}, \end{aligned}$$

with $C_B$ a constant depending only on *B*:

$$\begin{aligned} C_B=\left( \frac{3 W(\partial B)-2\pi \chi (\partial B) }{128\pi }\right) , \end{aligned}$$

where $W(\partial B)$ and $\chi (\partial B)$ denote, respectively, the Willmore energy and the Euler characteristic of the boundary surface $\partial B$.

##### Proof

(Proof of proposition 3)

Consider ${\widetilde{F}}\in H^S(\partial B)$. Since $\widetilde{{\mathbf {K}}}^*_B$ is a positive definite elliptic self-adjoint pseudo-differential operator of order $-1$ we can write [20, p. 290]:

$$\begin{aligned} H^{s}(\partial B) = \widetilde{{\mathbf {K}}}_B^*\left( H^{s-1}(\partial B)\right) \oplus \text {Ker\,}\left( \widetilde{{\mathbf {K}}}^*_B\right) , \end{aligned}$$

where $\text {Ker\,}(\widetilde{{\mathbf {K}}}^*_B)$ denotes the kernel of $\widetilde{{\mathbf {K}}}_B^*$. The symbol $\oplus$ is to be understood in the $L^2$ scalar product sense. Hence for $j\ge j_0$:

$$\begin{aligned} \left\langle {\widetilde{F}}, {\widetilde{\phi }}_j\right\rangle _{{\mathcal {H}}^*(\partial B)}&= - \left\langle {\widetilde{F}}, {\mathcal {S}}_B[ {\widetilde{\phi }}_j]\right\rangle _{L^2(\partial B)} \\&=- \left\langle {\widetilde{F}}, \left( -{\mathcal {S}}_B\right) ^{\frac{1}{2}}[ {\widetilde{\psi }}_j]\right\rangle _{L^2(\partial B)} \\&=- \left\langle \left( -{\mathcal {S}}_B\right) ^{\frac{1}{2}}[ {\widetilde{F}}],{\widetilde{\psi }}_j\right\rangle _{L^2(\partial B)}. \end{aligned}$$

where we used the fact that $\left( -{\mathcal {S}}_B\right) ^{\frac{1}{2}}$ is self-adjoint in $L^2(\partial B)$. Since $\left( -{\mathcal {S}}_B\right) ^{\frac{1}{2}}[ {\widetilde{F}}] \in H^{S+\frac{1}{2}}(\partial B)$ we have $\left( -{\mathcal {S}}_B\right) ^{\frac{1}{2}}[ {\widetilde{F}}]=\widetilde{{\mathbf {K}}}_B^*[{\widetilde{G}}^{(1)}] + {\widetilde{F}}^{(1)}_{\mathrm {ker}}$ with ${\widetilde{G}}^{(1)}\in H^{S-\frac{1}{2}}(\partial B)$. Then

$$\begin{aligned} \left\langle \left( -{\mathcal {S}}_B\right) ^{\frac{1}{2}}[ {\widetilde{F}}],{\widetilde{\psi }}_j\right\rangle _{L^2(\partial B)}= \,&\, \left\langle \widetilde{{\mathbf {K}}}_B^*[{\widetilde{G}}^{(1)}] + {\widetilde{F}}_{\mathrm {ker}}^{(1)} , {\widetilde{\psi }}_j\right\rangle _{L^2(\partial B)} \\ =\,&\, \lambda _j \left\langle {\widetilde{G}}^{(1)}, {\widetilde{\psi }}_j \right\rangle _{L^2(\partial B)} + \left\langle {\widetilde{F}}^{(1)}_{\mathrm {ker}} , {\widetilde{\psi }}_j\right\rangle _{L^2(\partial B)}. \end{aligned}$$

Since the eigenvectors of $\widetilde{{\mathbf {K}}}_B^*$ are orthogonal in $L^2(\partial B)$ we have:

$$\begin{aligned} \left\langle {\widetilde{F}}, {\widetilde{\phi }}_j\right\rangle _{{\mathcal {H}}^*(\partial B)} =- \lambda _j \left\langle {\widetilde{G}}^{(1)} ,{\widetilde{\psi }}_j\right\rangle _{L^2(\partial B)}. \end{aligned}$$

We can now write ${\widetilde{G}}^{(1)}= \widetilde{{\mathbf {K}}}_B^*[{\widetilde{G}}^{(2)}] + {\widetilde{F}}^{(2)}_{\mathrm {ker}}$ with ${\widetilde{G}}^{(2)}\in H^{S-\frac{3}{2}}(\partial B)$ and we have

$$\begin{aligned} \left\langle {\widetilde{G}}^{(1)} ,{\widetilde{\psi }}_j\right\rangle _{L^2(\partial B)} = \lambda _j \left\langle {\widetilde{G}}^{(2)} ,{\widetilde{\psi }}_j\right\rangle _{L^2(\partial B)}. \end{aligned}$$

Iterating this procedure $S-1$ times yields

$$\begin{aligned} \left\langle {\widetilde{G}}^{(1)} ,{\widetilde{\psi }}_j\right\rangle _{L^2(\partial B)} = \lambda _j^{S-1} \left\langle {\widetilde{G}}^{(S)} ,{\widetilde{\psi }}_j\right\rangle _{L^2(\partial B)}. \end{aligned}$$

Hence

13 $$\begin{aligned} \left\langle {\widetilde{F}}, {\widetilde{\phi }}_j\right\rangle _{{\mathcal {H}}^*(\partial B)} = -\lambda _j^S \left\langle {\widetilde{G}}^{(S)} ,{\widetilde{\psi }}_j\right\rangle _{L^2(\partial B)}. \end{aligned}$$

We need to control the $L^2$-norm of ${\widetilde{G}}^{(S)}$. We can rewrite the orthogonal decomposition as $\left( -{\mathcal {S}}_B\right) ^{\frac{1}{2}}[{\widetilde{F}}] = \left( \widetilde{{\mathbf {K}}}_B^*\right) ^S[{\widetilde{G}}^{(S)}]+ {\widetilde{F}}_{\mathrm {ker}}^{(1)}$. Composing by $\widetilde{{\mathbf {K}}}_B^*$ we get:

$$\begin{aligned} \widetilde{{\mathbf {K}}}_B^*\circ \left( -{\mathcal {S}}_B\right) ^{\frac{1}{2}}[ {\widetilde{F}}] = \left( \widetilde{{\mathbf {K}}}_B^*\right) ^{S+1}[{\widetilde{G}}^{(S)}]. \end{aligned}$$

Using the right-hand side of (12) with $s=S+\frac{1}{2}$ we get

$$\begin{aligned} \left\| \left( \widetilde{{\mathbf {K}}}_B^*\right) ^{S+1}[{\widetilde{G}}^{(S)}] \right\| _{H^{S+1}(\partial B)} \le C_{S+\frac{1}{2}} \left\| \left( -{\mathcal {S}}_B\right) ^{\frac{1}{2}}[ {\widetilde{F}}]\right\| _{H^S(\partial B)}. \end{aligned}$$

Using $S+1$ times the left hand side of (12) with $s-\frac{1}{2}=0,1,\ldots ,S$ yields

$$\begin{aligned} \left\| {\widetilde{G}}^{(S)}\right\| _{L^2(\partial B)}&\le \left( \prod _{s=0}^{S} \frac{1}{c_{s+\frac{1}{2}}} \right) \left\| \left( \widetilde{{\mathbf {K}}}_B^*\right) ^{S+1}[{\widetilde{G}}^{(S)}]\right\| _{H^{S+1}(\partial B)} \\&\le C_{S+\frac{1}{2}}\left( \prod _{s=0}^{S} \frac{1}{c_{s+\frac{1}{2}}} \right) \left\| \left( -{\mathcal {S}}_B\right) ^{\frac{1}{2}}[ {\widetilde{F}}]\right\| _{H^{S}(\partial B)}. \end{aligned}$$

Using the Cauchy–Schwartz inequality in (13) and the fact that $\Vert {\widetilde{\psi }}_j\Vert _{L^2(\partial B)}=1$ (${\mathcal {S}}_B$ is an isometry):

$$\begin{aligned} \left| \left\langle {\widetilde{F}}, {\widetilde{\phi }}_j\right\rangle _{{\mathcal {H}}^*(\partial B)} \right| \le C\lambda _j^S \Vert {\widetilde{F}} \Vert _{H^{S-\frac{1}{2}}(\partial B)}, \end{aligned}$$

where $C=C_{S+\frac{1}{2}}\left( \prod _{s=0}^{S} \frac{1}{c_{s+\frac{1}{2}}} \right)$ is independent of *j*. Using Theorem 2 we can see that for *j* large enough since $\lambda _j \sim C_B j^{-1/2}$ we have:

$$\begin{aligned} \left| \left\langle {\widetilde{F}}, {\widetilde{\phi }}_j\right\rangle _{{\mathcal {H}}^*(\partial B)} \right| \le j^{-S/2}C (C_B)^S \Vert {\widetilde{F}} \Vert _{H^{S-\frac{1}{2}}(\partial B)}, \end{aligned}$$

and since $j^{-S/2} C (C_B)^S = o\left( j^{-S/4}\right)$ we get the result. $\square$

#### The two-dimensional case

In two dimensions, the picture is slightly different. The eigenspace associated to eigenvalue zero can have infinite dimension (it is the case for the disk) and there are infinitely many negative eigenvalues. As a result, ${\mathcal {K}}_D^*$ can not be modified into a positive operator by adding a finite dimensional operator. However, for a certain class of domains, it is possible to show that there is a finite number of plasmonic resonances. For example, it was shown in [7] that an algebraic domain of class ${\mathcal {Q}}$ has asymptotically a finite number of plasmonic resonances. The asymptotic parameter is the deformation from the unit circle. For a larger class of domains the decay of the coefficients $\langle F, {\widetilde{\phi }}_j\rangle _{{\mathcal {H}}^*(\partial D)}$ can be checked numerically (see Sect. 6).

### Modal decomposition

Since the incoming wave is solution of the homogeneous Helmholtz equation in the background medium, standard elliptic regularity theory gives us $u^\text {in}\in C^\infty ({\mathbb {R}}^d)$. Moreover, the particle *B* is assumed to be $C^\infty$, so the source term in Eq. (6), i.e., the function ${\widetilde{F}}$, is smooth on $\partial B$. Therefore using Proposition 3 we have a super-polynomial decay of the coefficients $\langle {\widetilde{F}}, {\widetilde{\phi }}_j\rangle _{{\mathcal {H}}^*(\partial B)}$, and we can consider that only a finite number of modes are excited. The number *J* of modes to consider depends on the incoming field.

#### Proposition 4

Assume that ${\widetilde{F}}= \sum _{j=1}^J \left\langle {\widetilde{F}}, {\widetilde{\phi }}_j\right\rangle _{{\mathcal {H}}^*(\partial B)} {\widetilde{\phi }}_j$ on $\partial B$ for some $J\in {\mathbb {N}}^*$. The spectral approximation of the scattered field as $\omega \delta c^{-1}\rightarrow 0$ is given by

$$\begin{aligned} {\widetilde{u}}(X)-{\widetilde{u}}^\text {in}(X) = \sum _{j=0}^{J}\frac{1}{\tau _j(\omega )} \left\langle {\widetilde{F}},{\widetilde{\phi }}_j\right\rangle _{{\mathcal {H}}^*(\partial B)} \delta {\mathcal {S}}^{k_m\delta }_B[{\widetilde{\phi }}_j](X), \qquad X \in {\mathbb {R}}^d {\setminus } {\overline{B}}, \end{aligned}$$

where

$$\begin{aligned} \tau _j(\omega )= {\left\{ \begin{array}{ll} \tau _j+\left( \omega \delta c^{-1}\right) ^2 \log {\left( \omega \delta c^{-1}\right) }\tau _{j,1}+{\mathcal {O}}\left( \left( \omega \delta c^{-1}\right) ^2\right) , &{} d=2, \\ \tau _j+\left( \omega \delta c^{-1}\right) ^2\tau _{j,2}+{\mathcal {O}}\left( \left( \omega \delta c^{-1}\right) ^3\right) , &{} d=3, \end{array}\right. } \end{aligned}$$

with

$$\begin{aligned} \tau _{j,1}= \left\langle {\mathcal {A}}_{B,1}{\widetilde{\phi }}_j,{\widetilde{\phi }}_j\right\rangle _{{\mathcal {H}}^*(\partial B)}, \qquad \tau _{j,2}= \left\langle {\mathcal {A}}_{B,2}{\widetilde{\phi }}_j,{\widetilde{\phi }}_j\right\rangle _{{\mathcal {H}}^*(\partial B)}, \end{aligned}$$

and ${\widetilde{F}}$ is defined in Proposition 1.

#### Proof

Note that $\{{\widetilde{\phi }}_j\}_{j\in {\mathbb {N}}}$ forms an orthonormal basis of ${\mathcal {H}}^*(\partial B)$. Writing $\left( {\mathcal {A}}_B^0+{\mathcal {A}}^{\omega \delta c^{-1}}-{\mathcal {A}}_B^0\right) [{\widetilde{\varPsi }}]={\widetilde{F}}$ and using the decomposition of ${\widetilde{\varPsi }}$ in ${\mathcal {H}}^*(\partial B)$, ${\widetilde{\varPsi }}=\sum _{j=0}^{+\infty } \left\langle {\widetilde{\varPsi }},{\widetilde{\phi }}_j\right\rangle _{{\mathcal {H}}^*(\partial B)}{\widetilde{\phi }}_j$, yields the following:

$$\begin{aligned} \left\langle {\widetilde{\varPsi }},{\widetilde{\phi }}_j\right\rangle _{{\mathcal {H}}^*(\partial B)}= \left\{ \begin{aligned}&\frac{1}{\tau _j+\left\langle \left( {\mathcal {A}}^{\omega \delta c^{-1}} - {\mathcal {A}}_B^{0} \right) {\widetilde{\phi }}_j,{\widetilde{\phi }}_j\right\rangle _{{\mathcal {H}}^*(\partial B)}}\left\langle {\widetilde{F}},{\widetilde{\phi }}_j\right\rangle _{{\mathcal {H}}^*(\partial B)} \qquad&j\le J, \\&0 \qquad&j>J. \end{aligned} \right. \end{aligned}$$

Using (3) and (8) concludes the proof. $\square$

For each normalised eigenfunction of ${\mathcal {K}}^*_B$, we consider the corresponding function on $\partial D$,

$$\begin{aligned} \phi _j(x):={\widetilde{\phi }}_j\left( \frac{x-z}{\delta }\right) . \end{aligned}$$

Here $\{\phi _j\}_{j\in {\mathbb {N}}}$ are the rescaled non-normalised eigenfunctions of ${\mathcal {K}}_D^*$. Let us introduce

$$\begin{aligned} \varphi _j:=\frac{\phi _j}{||\phi _j||_{{\mathcal {H}}^*(\partial D)}}. \end{aligned}$$

Since $||{\widetilde{\phi }}_j||_{{\mathcal {H}}^*(\partial B)}=1$, we have (see Appendix B)

$$\begin{aligned} \varphi _j = {\left\{ \begin{array}{ll} \delta ^{-1} \phi _j, &{} d=2,\\ \delta ^{-3/2}\phi _j, &{} d=3. \end{array}\right. } \end{aligned}$$

Going back to the original unscaled problem:

#### Proposition 5

As $\omega \delta c^{-1}\ll 1$, the spectral decomposition of the field is as follows

14 $$\begin{aligned} u(x) =\left\{ \begin{aligned}&\sum _{j=0}^{J}\frac{1}{\tau _j(\omega )}\left\langle F,\varphi _j\right\rangle _{{\mathcal {H}}^*(\partial D)}{\mathcal {S}}^{k_m}_D[\varphi _j](x) + u^\text {in}(x),&x \in {\mathbb {R}}^d {\setminus } {\overline{D}},\\&\sum _{j=0}^{J}\frac{1}{\tau _j(\omega )}\left\langle F,\varphi _j\right\rangle _{{\mathcal {H}}^*(\partial D)}{\mathcal {S}}^{k_c}_D[\varphi _j](x),&x \in D.\ \end{aligned}\right. \end{aligned}$$

#### Proof

The scaling Lemma 11 gives ${\mathcal {S}}^{k_m\delta }_B[{\widetilde{\phi }}_j](X)=\delta ^{-1}{\mathcal {S}}^{k_m}_D[\phi _j](x)$ for $d=2,3$. From Lemma 12, we have $\langle {\widetilde{F}},{\widetilde{\phi }}_j \rangle _{{\mathcal {H}}^*(\partial B)}=\delta ^{-3}\left\langle F,\phi _j \right\rangle _{{\mathcal {H}}^*(\partial D)}$ for $d=3$ and $\langle {\widetilde{F}},{\widetilde{\phi }}_j \rangle _{{\mathcal {H}}^*(\partial B)}=\delta ^{-2}\left\langle F,\phi _j \right\rangle _{{\mathcal {H}}^*(\partial D)}$ for $d=2$. $\square$

## Plasmonic resonances

### Size dependent resonant frequencies

In this section we calculate size dependent plasmonic resonances. Let $j\in \{0,\ldots ,J\}$. Recall that

$$\begin{aligned} \tau _j(\omega )= {\left\{ \begin{array}{ll} \tau _j+\left( \omega \delta c^{-1}\right) ^2 \log {\left( \omega \delta c^{-1}\right) }\tau _{j,1}+{\mathcal {O}}\left( \left( \omega \delta c^{-1}\right) ^2\right) , &{} d=2, \\ \tau _j+\left( \omega \delta c^{-1}\right) ^2\tau _{j,2}+{\mathcal {O}}\left( \left( \omega \delta c^{-1}\right) ^3\right) , &{} d=3. \end{array}\right. } \end{aligned}$$

#### Definition 3

We say that $\omega$ is a static plasmonic resonance if $\left| \tau _j\right| =0$.

#### Definition 4

We say that $\omega$ is first-order corrected plasmonic resonance if

$$\begin{aligned} \left| \tau _j+(\omega \delta c^{-1})^2\log {(\omega \delta c^{-1})}\tau _{j,1}\right| =0 \end{aligned}$$

or

$$\begin{aligned} \left| \tau _j+(\omega \delta c^{-1})^2\tau _{j,2}\right| =0, \end{aligned}$$

with $d=2$ or $d=3$, respectively.

#### Remark 5

For $j=0$, we have $\tau _0=1/\varepsilon _m$, which is of size one by assumption. We exclude $j=0$ from the set of resonances.

For $j\ge 1$ we have ${\mathcal {P}}_{{\mathcal {H}}_0^*}[{\widetilde{\phi }}_j]={\widetilde{\phi }}_j$. Let us define

$$\begin{aligned} \alpha _j:= {\left\{ \begin{array}{ll} \left\langle \left( \frac{1}{2}I-{\mathcal {K}}^*_B\right) \widetilde{{\mathcal {S}}}_B^{-1}{\mathcal {S}}_{B,1}^{(1)}[{\widetilde{\phi }}_j], {\widetilde{\phi }}_j \right\rangle _{{\mathcal {H}}^*(\partial B)} , &{} d=2, \\ \left\langle \left( \frac{1}{2}I-{\mathcal {K}}^*_B\right) {\mathcal {S}}_B^{-1}{\mathcal {S}}_{B,2}[{\widetilde{\phi }}_j], {\widetilde{\phi }}_j \right\rangle _{{\mathcal {H}}^*(\partial B)} , &{} d=3. \end{array}\right. } \end{aligned}$$

Then, we can calculate

$$\begin{aligned} \tau _j(\omega )= {\left\{ \begin{array}{ll} \frac{\varepsilon _m-\varepsilon _c}{\varepsilon _m\varepsilon _c}\left( \lambda (\omega )-\lambda _j+\left( \omega \delta c^{-1}\right) ^2\log {\left( \omega \delta c^{-1}\right) }\alpha _j\right) +{\mathcal {O}}\left( \left( \omega \delta c^{-1}\right) ^2\right) , &{} d=2, \\ \frac{\varepsilon _m-\varepsilon _c}{\varepsilon _m\varepsilon _c}\left( \lambda (\omega )-\lambda _j+\left( \omega \delta c^{-1}\right) ^2\alpha _j\right) +{\mathcal {O}}\left( \left( \omega \delta c^{-1}\right) ^3\right) , &{} d=3. \end{array}\right. } \end{aligned}$$

#### Lemma 3

We have $\alpha _j \in {\mathbb {R}}$ and

$$\begin{aligned} \alpha _j:= {\left\{ \begin{array}{ll} \left( \lambda _j-\frac{1}{2} \right) \left\langle {\mathcal {S}}_{B,1}^{(1)} [{\widetilde{\phi }}_j], {\widetilde{\phi }}_j\right\rangle _{-1/2,1/2} , &{} d=2,\\ \left( \lambda _j-\frac{1}{2} \right) \left\langle {\mathcal {S}}_{B,2} [{\widetilde{\phi }}_j], {\widetilde{\phi }}_j\right\rangle _{-1/2,1/2}, &{} d=3. \end{array}\right. } \end{aligned}$$

In what follows we use the lower-case character $\omega$ for real frequencies and the upper-case character $\varOmega$ for complex frequencies.

#### Proposition 6

Using the Drude model (1), the three-dimensional first-order corrected plasmonic resonances $\varOmega _j^\pm (\delta ):=\pm \varOmega '_j+i\varOmega ''_j$ all lie in the lower part of the complex plane and their modulus is bounded. In the case where we take the medium to be vacuum, i.e., $\varepsilon _m=\varepsilon _0$ we obtain explicitly for $|\lambda _j+1/2| > 10^{-2}$ (this occurs, for example, when *B* is a ball [2]):

$$\begin{aligned} \varOmega _j' = \sqrt{\frac{\omega _p^2(\lambda _j+1/2)}{1+(\omega _p\delta c^{-1})^2\alpha _j} - \frac{ \mathrm {T}^{-2}}{4\left[ 1+\left( \omega _p \delta c^{-1}\right) ^2 \alpha _j\right] ^2}}\quad \text {and }\quad \varOmega _j'' = -\frac{\mathrm {T}^{-1}}{2\left[ 1+\left( \omega _p \delta c^{-1}\right) ^2 \alpha _j\right] }. \end{aligned}$$

Moreover, they are bounded

$$\begin{aligned} |\varOmega _j| \le 2\max \left\{ \frac{\mathrm {T}^{-1}}{\left| 1+\left( \omega _p\delta c^{-1}\right) ^2\alpha _j\right| },\frac{\omega _p\sqrt{\lambda _j+1/2}}{\sqrt{\left| 1+\left( \omega _p\delta c^{-1}\right) ^2\alpha _j\right| }}\right\} . \end{aligned}$$

#### Proof

We have that $\tau _j\left( \varOmega _j\right) = 0$ if and only if

$$\begin{aligned} \frac{\varOmega _j^2+i\varOmega _j \mathrm {T}^{-1}}{\omega _p^2} -\frac{1}{2} - \lambda _j + \frac{\varOmega _j^2\delta ^2}{c^2} \alpha _j=0, \end{aligned}$$

that is

$$\begin{aligned} {\left\{ \begin{array}{ll} \displaystyle \frac{1}{\omega _p^2}\left( \varOmega _j'^2-\varOmega _j''^2\right) - \frac{1}{2} - \lambda _j + \left( \varOmega _j'^2-\varOmega _j''^2\right) \frac{\delta ^2}{c^2} \alpha _j - \frac{1}{\omega _p^2\mathrm {T}}\varOmega _j''^2=0, \\ \displaystyle \frac{2}{\omega _p^2}\varOmega _j' \varOmega _j'' + 2\varOmega _j' \varOmega _j''\frac{\delta ^2 }{c^2} \alpha _j + \frac{1}{\omega _p^2 \mathrm {T}} \varOmega _j' =0. \end{array}\right. } \end{aligned}$$

Because $\delta \omega _p c^{-1} \ll 1$, we get the desired result. Lagrange improved upper-bound for roots of polynomials concludes the proof [27]. $\square$

#### Definition 5

In three dimensions, we define the resonance radius as

$$\begin{aligned} {\mathcal {R}}(\delta ):=\max _{j\in \{1,\ldots ,J\}}\max \left\{ \frac{2\mathrm {T}^{-1}}{\left| 1+\left( \omega _p\delta c^{-1}\right) ^2\alpha _j\right| },\frac{2\omega _p\sqrt{\lambda _j+1/2}}{\left| 1+\left( \omega _p\delta c^{-1}\right) ^2\alpha _j\right| ^{1/2}}\right\} . \end{aligned}$$

#### Remark 6

This resonance radius gives our method a range of validity. We compute resonant frequencies in a perturbative quasi–static regime. So by checking that

$$\begin{aligned} {\mathcal {R}}(\delta ) \delta c^{-1} < \frac{1}{2}, \end{aligned}$$

we ensure that the largest plasmonic frequency lies in a region that is still considered as *low-frequency* for a particle of size $\delta$. If we pick the size to be too large, namely such that ${\mathcal {R}}(\delta ) \delta c^{-1}$ is bigger than one, it means that the method is not self-consistent, as the largest resonant frequency might not satisfy the $\omega \delta c^{-1} <1/2$.

#### Proposition 7

In vacuum, and using the Drude model (1), the two-dimensional first-order corrected plasmonic resonances are the roots $\left( \varOmega _j\right) _{1\le j\le J}\in {\mathbb {C}}$ of the following equation

15 $$\begin{aligned} \frac{\varOmega _j^2+i\varOmega _j \mathrm {T}^{-1}}{\omega _p^2} -\frac{1}{2} - \lambda _j +\left( \varOmega _j\delta c^{-1}\right) ^2 \log {\left( \varOmega _j\delta c^{-1}\right) } \alpha _j=0. \end{aligned}$$

#### Remark 7

We can compute an approximation of the roots of (15) by computing in the first place the static resonances $\left( \varOmega _{s,j}\right) _{1\le j\le J}$. Solving $\tau _j=0$ yields

$$\begin{aligned} \varOmega ^\pm _{s,j}=\pm \sqrt{\omega _p^2\left( \lambda _j+\frac{1}{2}\right) - \frac{1}{4T^2}} - \frac{i}{2T}. \end{aligned}$$

Replacing the dynamic frequency in the logarithm by its static approximation, we transform (15) into the quadratic equation

$$\begin{aligned} \frac{\varOmega _j^2+i\varOmega _j \mathrm {T}^{-1}}{\omega _p^2} -\frac{1}{2} - \lambda _j +\left( \varOmega _j\delta c^{-1}\right) ^2 \log {\left( \varOmega _{s,j}\delta c^{-1}\right) } \alpha _j=0. \end{aligned}$$

We get

16 $$\begin{aligned} \varOmega _j^\pm (\delta )=\frac{-i\mathrm {T}^{-1}\pm \sqrt{4\omega _p^2\left( \lambda _j+1/2\right) \left[ 1+\alpha _j\left( \omega _p\delta c^{-1}\right) ^2\log {\left( \varOmega ^\pm _{s,j}\delta c^{-1}\right) }\right] }}{2\left[ 1+\alpha _j\left( \omega _p\delta c^{-1}\right) ^2\log {\left( \varOmega ^\pm _{s,j}\delta c^{-1}\right) }\right] }. \end{aligned}$$

#### Definition 6

In two dimensions, we define the resonance radius as

$$\begin{aligned} {\mathcal {R}}(\delta )&:=\max _{j\in \{1,\ldots ,J\}}\max \left\{ \frac{2\mathrm {T}^{-1}}{\left| 1+\alpha _j\left( \omega _p\delta c^{-1}\right) ^2\log {\left( \varOmega ^\pm _{s,j}\delta c^{-1}\right) }\right| }, \right. \\&\quad \left. \frac{2\omega _p\sqrt{\lambda _j+1/2}}{\left| 1+\alpha _j\left( \omega _p\delta c^{-1}\right) ^2\log {\left( \varOmega ^\pm _{s,j}\delta c^{-1}\right) }\right| ^{1/2}}\right\} . \end{aligned}$$

### Plasmonic quasi-normal modes

Quasi-normal modes are formally defined as solutions of the source-free wave equation [28]. Using the representation formula (3), we can now define, as in the physics literature, plasmonic quasi-normal modes $(e_j^{\pm })_{j\in {\mathbb {N}}}$ that oscillate at complex frequencies $\varOmega _j^{\pm }(\delta )$:

17 $$\begin{aligned} e_j^{\pm }(x)= \left\{ \begin{aligned}&{\mathcal {S}}^{\varOmega _j^{\pm }(\delta )c^{-1}}_D[\varphi _j](x),&x\in {\mathbb {R}}^d{\setminus } {\overline{D}},\\&{\mathcal {S}}^{\varOmega _j^{\pm }(\delta )\sqrt{\varepsilon _c \mu _m}}_D[\varphi _j](x),&x\in D. \end{aligned}\right. \end{aligned}$$

These $(e_j^{\pm })_{j\in {\mathbb {N}}}$ solve the source-free Helmholtz equation and satisfy the radiation condition at infinity, but they diverge exponentially fast as $\vert x\vert \rightarrow \infty$.

#### Remark 8

In the physics literature (see [28, equation (1.1)] for instance) one can often find representations of the scattered field in the form

$$\begin{aligned} u(x,\omega ) = \sum _j \alpha _j(u^\text {in}, \omega ) e_j^\pm (x), \end{aligned}$$

where $\alpha _j$ are *excitation coefficients* depending on the source and independent of the space variable *x*. The first problem of these representations is that the eigenmodes diverge exponentially and a renormalisation process is necessary. Numerical instability will appear far away from the particle. Even though the study of these modes *individually* can give physical insight to a system (like for example by studying the mode volume quantity [16]), they cannot be used in frequency domain representation formulae to solve the scattering problem. Moreover, this type of representation formulae is a generalisation of the well-known eigenmode expansions for waves in bounded domains with classical Dirichlet or Neumann conditions. For these formulae to be valid one needs to address the question of completeness of the eigenbasis. Here we have shown the completeness of the charge distribution modes on the boundary of the particle only. Moreover, our perturbative analysis only holds for a finite number of modes and cannot be used to show completeness of the perturbed modes as soon as $\omega \not = 0$.

## Time domain approximation of the scattered field

In the following section we show that even though they are irrelevant for frequency domain representation, quasi-normal modes can be used to approximate the field in the time domain. The idea is to get around costly time domain computations by pre-computing the modes of the system and then expressing the response of the system to any source in terms of the modes. In the physics literature (for example [28, eq. (1.2)]) the field in the time domain is expressed under the form

18 $$\begin{aligned} u(x,t)= \mathfrak {R}\, \sum _j \beta _j(t) e_j^\pm (x). \end{aligned}$$

The problem with this type of expansions is that if $\vert x \vert$ is big then $e_j^\pm (x)$ is exponentially large and the computation of *u*(*x*, *t*) is not very stable if the modes are pre-computed.

We will show in this section that it is possible to express the scattered field in the time domain in a similar expansion, but with non-diverging, pre-computable quantities similar to the quasi-normal modes.

### The three-dimensional case

Here we state the main result of the paper, Theorem 3, and discuss the result.

#### The modal approximation

Let $\varGamma ^{k_m}(\cdot ,s)$, i.e., the Green’s function for the Helmholtz equation introduced in Definition 7, be the incident wave $u^\text {in}$ in three dimensions. Given a wideband signal ${\widehat{f}}:t \mapsto {\widehat{f}}(t) \in C_0^{\infty }([0,C_1])$, for $C_1>0$, we want to express the time domain response of the electric field to an oscillating dipole placed at a source point *s*. This means that for a fixed $\delta$ we can pick an excitation signal such that most of the frequency content is in the *low frequencies* but large enough to excite the plasmonic resonances. We can pick $\eta \ll 1$ and $\rho \ge {\mathcal {R}}(\delta )$ such that

$$\begin{aligned} \int _{{\mathbb {R}}{\setminus }[-\rho ,\rho ]} \vert f(\omega ) \vert ^2 \mathrm {d}\omega&\le \eta , \\ \frac{\rho \delta }{c}&\le 1, \end{aligned}$$

where $f:\omega \mapsto f(\omega )$ is the Fourier transform of ${\widehat{f}}$. In practice we take $\rho = {\mathcal {R}}(\delta )$. The incident field has the following form in the time domain:

$$\begin{aligned} {\widehat{u}}^\text {in}(x,t)=\int _{{\mathbb {R}}} \varGamma ^{\frac{\omega }{c}}(x,s) f(\omega )e^{-i\omega t} \mathrm {d}\omega =\frac{{\widehat{f}}(t-|x-s|/c)}{4\pi |x-s|}. \end{aligned}$$

The goal of this section is to establish a resonance expansion for the low-frequency part of the scattered electric field in the time domain. Introduce, for $\rho >0$, the truncated inverse Fourier transform of the scattered field $u^{\text {sca}}$ given by

$$\begin{aligned} P_\rho \left[ u^\text {sca}\right] (x,t):=\int _{-\rho }^{\rho } u^{\text {sca}}(x,\omega ) e^{-i\omega t} \mathrm {d}\omega . \end{aligned}$$

Recall that *z* is the centre of the resonator and $\delta$ its radius. Let us define

$$\begin{aligned} t_0^\pm (s,x):=\frac{1}{c}\left( |s-z|+|x-z|\pm 2\delta \right) \pm C_1, \end{aligned}$$

the time it takes to the wideband signal to reach first the scatterer and then the observation point *x*. The term $\pm 2 \delta /c$ accounts for the maximal timespan spent inside the particle.

Recall the spectral decomposition in the frequency domain (Proposition 5) for $x \in {\mathbb {R}}^3{\setminus } {\overline{D}}$:

$$\begin{aligned} u^\text {sca}(x,\omega )=\left( u-u^\text {in}\right) (x,\omega )=\sum _{j=1}^{J}\frac{1}{\tau _j(\omega )}\left\langle F,\varphi _j\right\rangle _{{\mathcal {H}}^*(\partial D)}{\mathcal {S}}^{\frac{\omega }{c}}_D[\varphi _j](x). \end{aligned}$$

##### Theorem 3

Let $N\in {\mathbb {N}}$. The scattered field has the following form in the time domain for $x\in {\mathbb {R}}^3{\setminus } {\overline{D}}$:

19 $$\begin{aligned} P_{\rho }\left[ u^\text {sca}\right] (x,t)= {\left\{ \begin{array}{ll} {\mathcal {O}}\left( \delta ^4 \rho ^{-N}\right) , &{} t\le t_0^-, \\ 2\pi i\sum _{j=1}^J C_{\varOmega _j^\pm (\delta )} \langle F, \varphi _j \rangle _{{\mathcal {H}}^*(\partial D)} e_j^\pm (x) e^{-i\varOmega _j^\pm (\delta ) t}+{\mathcal {O}}\left( \frac{\delta ^4}{t}\rho ^{-N}\right) , &{} t\ge t_0^+. \end{array}\right. } \end{aligned}$$

The complex numbers $\varOmega _j^\pm (\delta )$ are the resonant frequencies given by Proposition 6. The fields $e_j$ are the classical quasi-normal modes defined in Sect. 4.2. $C_{\varOmega _j^\pm (\delta )}$ is a constant depending only on *j*, the size $\delta$ and the model for $\varepsilon _c(\omega )$:

$$\begin{aligned} C_{\varOmega _j^\pm (\delta )}:=\varepsilon _0 \frac{\left( {\varOmega ^\pm _j(\delta )}^2 + i \varOmega ^\pm _j(\delta ) T^{-1} -\omega _p^2\right) }{\left( 1+\left( \omega _p\delta c^{-1}\right) ^2 \alpha _j\right) \left( \varOmega ^\pm _j(\delta )-\varOmega ^\mp _j(\delta )\right) } . \end{aligned}$$

##### Remark 9

The resonant frequencies $\left\{ \varOmega _j^\pm (\delta )\right\} _{1\le j\le J}$ have negative imaginary parts, so Theorem 3 expresses the scattered field as the sum of decaying oscillating fields. The imaginary part of $\varOmega _j^\pm (\delta )$ accounts for absorption losses in the particle as well as radiative losses.

##### Remark 10

(About the remainder $\rho$) Since for a particle of finite size $\delta$ our expansion only holds for a range of frequencies $\omega$ such that $\omega \delta c^{-1} <1$, we cannot compute the full inverse Fourier transform and we have a remainder that depends on the maximum frequency that we can use. Nevertheless that maximum frequency $\rho$ behaves as $c/\delta$ and we can see that the remainder gets arbitrarily small for small particles. For a completely point-like particle one would get a zero remainder.

##### Remark 11

If we had access to the full inverse Fourier transform of the field, of course, since the inverse Fourier transform of a function which is analytic in the upper-half plane is *causal* we would find that in the case $t\le \left( |s-z|+|x-z|-2\delta \right) /c$, ${\widehat{u}}^{\text {sca}}(x,t)= 0$. Nevertheless, our method gives the resonant frequencies only in the *low-frequency* regime. Therefore we only have an approximation for the *low-frequency* part of the scattered field, which does not have a compact support in time. Nevertheless, as shown in the numerical section 6.4.5, the low-frequency part of the scattered field is actually a good approximation for the scattered field. There does not seem to be any resonant frequencies for $\omega > {\mathcal {R}}(\delta )$. This is highly non-trivial and we do not have a mathematical justification for that. Physically though, it can be explained by looking at the Drude model and noting that when $\omega \rightarrow \infty$, $\varepsilon (\omega )\longrightarrow 1$. The metal does not really interact with light at high frequencies.

#### Alternative formulation with non-diverging causal quasi-normal modes

Even though $\vert e_j^\pm (x)\vert \longrightarrow \infty$ when $\vert x\vert \rightarrow \infty$, no terms diverge in (19). Indeed we can rewrite:

$$\begin{aligned} e_j^\pm (x) e^{-i\varOmega _j^\pm (\delta ) t}&= e_j^\pm (x)e^{-i\varOmega _j^\pm (\delta ) t_0^+} e^{-i\varOmega _j^\pm (\delta ) (t-t_0)}\\&= e_j^\pm (x)e^{-i\varOmega _j^\pm (\delta ) \left( |s-z|+|x-z|+ 2\delta \right) c^{-1}+ C_1} e^{-i\varOmega _j^\pm (\delta ) (t-t_0^+)}\\&= C_{u^\text {in}, \delta } e_j^\pm (x) e^{-i\varOmega _j^\pm (\delta ) |x-z|c^{-1}}e^{-i\varOmega _j^\pm (\delta ) (t-t_0^+)}, \end{aligned}$$

where $C_{u^\text {in}, \delta }$ depends only on the incoming field and the particle size. We can define the following *causal plasmonic quasi-normal modes* $(E_j^\pm )_{j\in {\mathbb {N}}}$ at the complex frequency $\varOmega _j^\pm (\delta )$:

20 $$\begin{aligned} E_j^{\pm }(x)= \left\{ \begin{aligned}&{\mathcal {S}}^{\varOmega ^\pm _j(\delta ) c^{-1}}_D[\varphi _j](x)e^{-i\varOmega _j^\pm (\delta ) |x-z|c^{-1}},&x\in {\mathbb {R}}^d{\setminus } {\overline{D}},\\&{\mathcal {S}}^{\varOmega ^\pm _j(\delta )\sqrt{\varepsilon _c \mu _m}}_D[\varphi _j](x),&x\in D. \end{aligned}\right. \end{aligned}$$

##### Remark 12

When referring to $E_j^\pm$, the term *mode* is inaccurate, as $E_j^\pm$ does not solve the Helmholtz equation. But since the $(E_j^\pm )_{j\in {\mathbb {N}}}$ are built from modes with a complex phase correction, we still call them modes in a loose sense of the term.

Theorem 3 can be re-stated:

##### Theorem 4

(Alternative causal expansion)

21 $$\begin{aligned} P_{\rho }\left[ u^\text {sca}\right] (x,t)= {\left\{ \begin{array}{ll} {\mathcal {O}}\left( \delta ^4 \rho ^{-N}\right) , &{} t\le t_0^-, \\ 2\pi i\sum _{j=1}^J \beta _j^{\delta }(u^\text {in}) E_j^\pm (x) e^{-i\varOmega _j^\pm (\delta ) (t-t_0^+)}+{\mathcal {O}}\left( \frac{\delta ^4}{t}\rho ^{-N}\right) , &{} t\ge t_0^+, \end{array}\right. } \end{aligned}$$

where $$ \beta _j^{\delta }(u^\text {in}) = C_{\varOmega _j^\pm (\delta )} \langle F, \varphi _j \rangle _{{\mathcal {H}}^*(\partial D)}e^{-i\varOmega _j^\pm (\delta ) \left( |s-z|+ 2\delta \right) c^{-1}+ C_1}.$$

##### Remark 13

Expansion (21) has exactly the same form as the representation formula found in the physics literature (like Eq. (18)) but without any exponentially diverging quantities. The $E_j^\pm$ can be computed independently of the source, just like regular quasi-normal modes.

### Proof of Theorem 3

Before we can prove Theorem 3 we need the following lemma:

#### Lemma 4

As $\omega \delta c^{-1} \rightarrow 0$, *F* defined in (7) admits the following asymptotic expansion:

22 $$\begin{aligned} F(x) = \frac{1}{\delta }\left[ \delta \left( \frac{1}{\varepsilon _c}-\frac{1}{\varepsilon _m}\right) \nu _x\cdot \nabla \varGamma ^{k_m}(z-s)+{\mathcal {O}}\left( \left( \omega \delta c^{-1}\right) ^2\right) \right] , \quad x \in \partial D. \end{aligned}$$

#### Proof

See Appendix C.3. $$\square $$

#### Proof

(Proof of Theorem 3) We start by studying the time domain response of a single mode to a causal excitation at the source point *s*. According to Proposition 5 we need to compute the contribution $\varXi _j$ of each mode, that is,

$$\begin{aligned}&\int _{-\rho }^{\rho }\varXi _j(x,\omega ) e^{-i\omega t}\mathrm {d}\omega \\&\quad :=\int _{-\rho }^\rho \frac{1}{\lambda (\omega )-\lambda _j(\omega \delta )}\left\langle \nabla \varGamma ^{\frac{\omega }{c}}(z ,s)\cdot \nu (\cdot ) f(\omega ),\varphi _j \right\rangle _{{\mathcal {H}}^*(\partial D)} {\mathcal {S}}_D^{\frac{\omega }{c}}\left[ \varphi _j\right] e^{-i\omega t}\mathrm {d}\omega , \end{aligned}$$

where $\lambda _j(\omega \delta ) := \lambda _j - \left( \omega \delta c^{-1}\right) ^2 \alpha _{j} +{\mathcal {O}}\left( \left( \omega \delta c^{-1}\right) ^3\right)$. One can then write:

$$\begin{aligned}&\left\langle \nabla \varGamma ^{\frac{\omega }{c}}(z ,s)\cdot \nu (\cdot ) f(\omega ),\varphi _j \right\rangle _{{\mathcal {H}}^*(\partial D)} {\mathcal {S}}_D^{\frac{\omega }{c}}\left[ \varphi _j\right] \\&\quad = f(\omega ) \left( \frac{1}{|z-s|}-i\frac{\omega }{c}\right) \left( \lambda _j-\frac{1}{2}\right) \int _{\partial D\times \partial D} \frac{v\varphi _j(v)\varphi _j(y)}{16\pi ^2|x-y||z-s|}e^{i\frac{\omega }{c}(|x-y|+|z-s|)}\mathrm {d}\sigma (v) \mathrm {d}\sigma (y), \end{aligned}$$

where we used $\left\langle \nu ,\varphi _j\right\rangle _{{\mathcal {H}}^*(\partial D)}=(1/2-\lambda _j)\left\langle x,\varphi _j\right\rangle _{\frac{1}{2},-\frac{1}{2}}$ [6]. Since $\left\langle \nu ,\varphi _0\right\rangle _{{\mathcal {H}}^*(\partial D)}=0$, the zeroth term vanishes in the summation.

Now we want to apply the residue theorem to get an asymptotic expansion in the time domain. Note that:

$$\begin{aligned} \int _{-\rho }^{\rho } \varXi _j(x,\omega ) e^{-i\omega t} \mathrm {d}\omega = \oint _{{\mathcal {C}}^{\pm }} \varXi _j(x,\varOmega ) e^{-i\varOmega t}\mathrm {d}\varOmega -\int _{{\mathcal {C}}_\rho ^{\pm }} \varXi _j(x,\varOmega ) e^{-i\varOmega t} \mathrm {d}\varOmega , \end{aligned}$$

where the integration contour ${\mathcal {C}}_\rho ^{\pm }$ is a semicircular arc of radius $\rho$ in the upper (+) or lower (−) half-plane, and ${\mathcal {C}}^{\pm }$ is the closed contour ${\mathcal {C}}^{\pm }={\mathcal {C}}_\rho ^{\pm }\cup [-\rho ,\rho ]$. The integral on the closed contour is the main contribution to the scattered field by the mode and can be computed using the residue theorem to get, for $\rho \ge \mathfrak {R}[\varOmega _j^\pm (\delta )]$,

$$\begin{aligned} \oint _{{\mathcal {C}}^{+}} \varXi _j(x,\varOmega ) e^{-i\varOmega t} \mathrm {d}\varOmega&=0,\\ \oint _{{\mathcal {C}}^{-}} \varXi _j(x,\varOmega ) e^{-i\varOmega t} \mathrm {d}\varOmega&=2\pi i\text {Res}\left( \varXi _j(x,\varOmega )e^{-i\varOmega t},\varOmega _j^\pm (\delta )\right) . \end{aligned}$$

Since $\varOmega _j^\pm (\delta )$ is a simple pole of $\omega \mapsto \dfrac{1}{\lambda (\omega )-\lambda _j(\omega \delta )}$ we can write:

$$\begin{aligned} \oint _{{\mathcal {C}}^{-}} \varXi _j(x,\varOmega ) e^{-i\varOmega t} \mathrm {d}\varOmega&=2\pi i\text {Res}\left( \varXi _j(x,\varOmega ),\varOmega _j^\pm (\delta )\right) e^{-i\varOmega _j^\pm (\delta )t}. \end{aligned}$$

To compute the integrals on the semi-circle, we introduce:

$$\begin{aligned} B_j(y,v,\varOmega )=\frac{\lambda _j-1/2}{\lambda (\omega )-\lambda _j(\delta \varOmega )}\left( \frac{1}{|z-s|}-i\frac{\omega }{c}\right) \int _{\partial D\times \partial D} \frac{v\varphi _j(v)\varphi _j(y)}{16\pi ^2|x-y||z-s|} \qquad (y,v) \in (\partial D)^2. \end{aligned}$$

Note that $B_j(\cdot ,\cdot ,\varOmega )$ behaves like a polynomial in $\varOmega$ when $\vert \varOmega \vert \rightarrow \infty$. Given the regularity of the input signal ${\widehat{f}} \in C_0^{\infty }([0,C_1])$, the Paley–Wiener theorem [46, p. 161] ensures decay properties of its Fourier transform at infinity. For all $N\in {\mathbb {N}}^*$ there exists a positive constant $C_N$ such that for all $\varOmega \in {\mathbb {C}}$

$$\begin{aligned} |f(\varOmega )|\le C_N (1+|\varOmega |)^{-N}e^{C_1 |\mathfrak {I}{(\varOmega )}|}. \end{aligned}$$

Let $T:=(\vert x-y\vert + \vert s-v\vert )/c$. We now re-write the integrals on the semi-circle:

$$\begin{aligned} \int _{{\mathcal {C}}_\rho ^{\pm }} \varXi _j(x,\varOmega ) e^{-i\varOmega t} \mathrm {d}\varOmega =\int _{{\mathcal {C}}_\rho ^{\pm }}f(\varOmega ) \int _{\partial D\times \partial D} B_j(y,v,\varOmega ) e^{i \varOmega \left( T -t\right) }\mathrm {d}\sigma (v)\mathrm {d}\sigma (y) \mathrm {d}\varOmega . \end{aligned}$$

We have that $t_0^-+C_1 \le T \le t_0^+-C_1$. Two cases arise.

*Case 1:* For $0<t<t_0^-$ , i.e., when the signal emitted at *s* has not reached the observation point *x*, we choose the upper-half integration contour ${\mathcal {C}}^+$. Transforming into polar coordinates, $\varOmega =\rho e^{i\theta }$ for $\theta \in [0,\pi ]$, we get:

$$\begin{aligned} \left| e^{i \varOmega \left( T -t\right) }\right| \le e^{ -(t_0^--t+C_1)\mathfrak {I}(\varOmega )} \qquad \forall (y,v)\in (\partial D)^2, \end{aligned}$$

and

$$\begin{aligned}&\left| \int _{{\mathcal {C}}_\rho ^{+}} \varXi _j(x,\varOmega ) e^{-i\varOmega t} \mathrm {d}\varOmega \right| \\&\quad \le \int _0^\pi \rho \left| f\left( \rho e^{i\theta }\right) \right| e^{-\rho (t_0^--t+C_1)\sin {\theta }}\int _{\partial D \times \partial D}\left| B_j\left( y,v,\rho e^{i\theta }\right) \right| \mathrm {d}\sigma (v) \mathrm {d}\sigma (y) \mathrm {d}\theta ,\\&\quad \le \rho C_N(1+\rho )^{-N}\delta ^4 \max _{\theta \in [0,\pi ]}{\left\| B_j\left( \cdot , \cdot , \rho e^{i\theta }\right) \right\| _{L^{\infty }(\partial D\times \partial D)}} \pi \frac{1-e^{-\rho (t^-_0-t)}}{\rho (t^-_0-t)}, \end{aligned}$$

where we used that for $\theta \in [0,\pi /2]$, we have $\sin {\theta } \ge 2\theta /\pi \ge 0$ and $-\cos {\theta }\le -1+2\theta /\pi$. The usual way to go forward from here is to take the limit $\rho \rightarrow \infty$, and get that the limit of the integral on the semi-circle is zero. However, we work in the quasi–static approximation here, and our modal expansion is not uniformly valid for all frequencies. So we have to work with a fixed maximum frequency $\rho$. Since *N* can be taken arbitrarily large and that $B_j$ behaves like a polynomial in $\rho$ *whose degree does not depend on j*, we get that, uniformly for $j\in [1,J]$:

$$\begin{aligned} \left| \int _{{\mathcal {C}}_\rho ^{+}} \varXi _j(x,\varOmega ) e^{-i\varOmega t} \mathrm {d}\varOmega \right| = {\mathcal {O}}\left( \frac{\delta ^4}{t_0^--t} \rho ^{-N}\right) . \end{aligned}$$

Of course if one has to consider the full inverse Fourier transform of the scattered electromagnetic field, by causality, one should expect the limit to be zero. However, one would need high-frequency estimates of the electromagnetic field, as well as a modal decomposition that is uniformly valid for all frequencies. Since our modal expansion is only valid for a limited range of frequencies we get an error bound that is arbitrarily small if the particle is arbitrarily small, but not strictly zero.

*Case 2:* For $t>t_0^+$, we choose the lower-half integration contour ${\mathcal {C}}^-$. Transforming into polar coordinates, $\varOmega =\rho e^{i\theta }$ for $\theta \in [\pi ,2\pi ]$, we get

$$\begin{aligned} \left| e^{i \varOmega \left( T -t\right) }\right| \le e^{ (t-t_0^+-C_1) \mathfrak {I}(\varOmega )} \qquad \forall (y,v)\in (\partial D)^2, \end{aligned}$$

and

$$\begin{aligned}&\left| \int _{{\mathcal {C}}_\rho ^{-}} \varXi _j(x,\varOmega ) e^{-i\varOmega t} \mathrm {d}\varOmega \right| \\&\quad \le \int _\pi ^{2\pi }\rho \left| f\left( \rho e^{i\theta }\right) \right| e^{\rho (t-t_0^+-C_1)\sin {\theta }}\int _{\partial D\times \partial D}\left| B_j\left( y,v,\rho e^{i \theta }\right) \right| \mathrm {d}\sigma (v) \mathrm {d}\sigma (y) \mathrm {d}\theta ,\\&\quad \le \rho C_N(1+\rho )^{-N} \delta ^4 \max _{\theta \in [\pi ,2\pi ]}{\left\| B_j\left( \cdot , \cdot , \rho e^{i\theta }\right) \right\| _{L^{\infty }(\partial D\times \partial D)}}\pi \frac{1-e^{-\rho (t-t_0^+)}}{\rho (t-t_0^+)}. \end{aligned}$$

Exactly as in Case 1, we cannot take the limit $\rho \rightarrow \infty$. Using the fact that *N* can be taken arbitrarily large and that $B_j$ behaves like a polynomial in $\rho$ *whose degree does not depend on j*, we get that, uniformly for $j\in [1,J]$:

$$\begin{aligned} \left| \int _{{\mathcal {C}}_\rho ^{-}} \varXi _j(x,\varOmega ) e^{-i\varOmega t} \mathrm {d}\varOmega \right| = {\mathcal {O}}\left( \frac{\delta ^4}{t} \rho ^{-N}\right) . \end{aligned}$$

The result of Theorem 3 is obtained by summing the contribution of all the modes considered. $\square$

#### Remark 14

The fact that we work with a finite number of modes is necessary for the perturbation theory of Sect. 3 but also in this section. Indeed, if we consider all the modes there is an accumulation point in the poles of the modal expansion of the field, and therefore we cannot apply the residue theorem.

### The two-dimensional case

In two dimensions, the Green’s function does not have an explicit phase term, so we need to introduce another asymptotic parameter $\epsilon >0$ to be able to use the large argument asymptotics of the Hankel function. Our new truncated inverse Fourier transform of the scattered field $u^{\text {sca}}$ given by

$$\begin{aligned} P_{\rho ,\epsilon }\left[ u^\text {sca}\right] (x,t)=\int _{-\rho }^{-\epsilon } u^{\text {sca}}(x,\omega ) e^{-i\omega t} \mathrm {d}\omega +\int _{\epsilon }^{\rho } u^{\text {sca}}(x,\omega ) e^{-i\omega t} \mathrm {d}\omega . \end{aligned}$$

This allows us to define a notion of *far field*. A point *x* is far from *D* if $\epsilon \vert x-z\vert c^{-1}\gg 1$. We can now add two additional hypotheses:

- the source is far away from the particle (or equivalently, the incoming wave is a plane wave)
- the observation point is far away from the particle.

The incident field has the following form in the time domain:

23 $$\begin{aligned} {\widehat{u}}^{\text {in}}(x,t)={\widehat{f}}\left( t-\frac{d\cdot x}{c}\right) . \end{aligned}$$

Besides these two assumptions and a difference in the order of the remainder, the result in two dimensions is essentially the same as in three dimensions.

#### Theorem 5

Let $N\in {\mathbb {N}}$. The scattered field has the following form in the time domain for *x* far away from *D*:

24 $$\begin{aligned} P_{\rho ,\epsilon }\left[ u^\text {sca}\right] (x,t)= {\left\{ \begin{array}{ll} {\mathcal {O}}\left( \delta \rho ^{-N}\right) , &{} t\le t_0^-, \\ 2\pi i\sum _{j=1}^J C_{\varOmega _j^\pm (\delta )} \langle F, \varphi _j \rangle _{{\mathcal {H}}^*(\partial D)} e_j^\pm (x) e^{-i\varOmega _j^\pm (\delta ) t}+{\mathcal {O}}\left( \frac{\delta }{t}\rho ^{-N}\right) , &{} t\ge t_0^+, \end{array}\right. } \end{aligned}$$

with $\varOmega _j^\pm (\delta )$ being the plasmonic resonant frequencies of the particle given by Proposition 7. $C_{\varOmega _j^\pm (\delta )}$ is a constant depending only on *j*, the size $\delta$ and the model for $\varepsilon _c(\omega )$:

$$\begin{aligned} C_{\varOmega _j^\pm (\delta )}:=\varepsilon _0 \frac{\left( {\varOmega ^\pm _j(\delta )}^2 + i \varOmega ^\pm _j(\delta ) T^{-1} -\omega _p^2\right) }{\left( 1+\left( \omega _p\delta c^{-1}\right) ^2 \log \left( \varOmega ^\pm _{s,j}\delta c^{-1}\right) \alpha _j\right) \left( \varOmega ^\pm _j(\delta )-\varOmega ^\mp _j(\delta )\right) } . \end{aligned}$$

#### Proof

The proof is quite similar to the three-dimensional case. It is included in Appendix D for the sake of completeness. $\square$

## Numerical simulations

The goal of this section is to illustrate the validity of our approach and to show that the approximation seems to be working with less restrictive hypotheses than the ones in Theorem 5:

- for more general shapes (non-convex or non-algebraic)
- closer to the particle (outside of the far-field approximation).

For these simulations we build upon the codes for layer potentials developed in [44].

### Domains and physical parameters

**Fig. 1:**
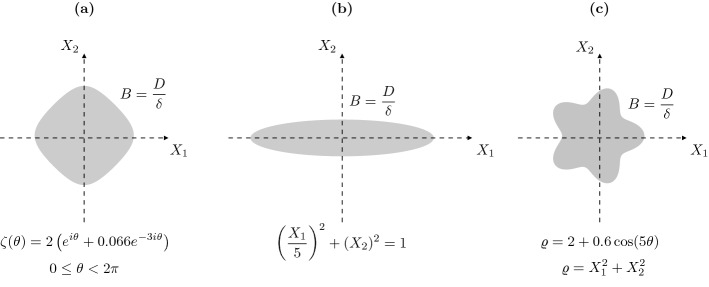
Sketch of the three reference domains: the rounded diamond (**a**), the narrow ellipse (**b**) and the five-petal flower (**c**)

Throughout this section, we consider the three domains sketched on Fig. 1 to illustrate our results:

*Rounded diamond:*

The rounded diamond (a) is defined by the parametric curve $\zeta (\theta )=2\left( e^{i\theta }+0.066e^{-3i\theta }\right)$, for $\theta \in [0,2\pi ]$. It is an algebraic domain of class ${\mathcal {Q}}$ from [7]. This shape satisfies condition 1, as well as the hypotheses of Theorem 5.

*Narrow ellipse:*

The ellipse (b) semi-axes are on the $X_1$- and $X_2$-axes and are of length $a=1$ and $b=5$, respectively. It is algebraic but not asymptotically a circle in the sense of [7].

*Five-petal flower:*

The flower (c) is defined by $\varrho =2+0.6\cos (5\theta )$ in polar coordinates. It has Cartesian equation

$$\begin{aligned} 0.5\left( X_1^2+X_2^2\right) ^3-1.5X_1\left( X_1^2+X_2^2\right) ^2+6X_1^3\left( X_1^2+X_2^2\right) -4.8X_1^5\left( X_1^2+X_2^2\right) ^3-\left( X_1^2+X_2^2\right) ^{5/2}=0 \end{aligned}$$

in the rescaled $(X_1,X_2)$ plane. So it is not algebraic (due to the non-integer power of the last term) and not convex. We have no theoretical results on the number of modes that radiate.

All three domains $D=z+\delta B$ are centred at the origin $(z=0)$ for simplicity. We set the size of the nanoparticle to be $\delta =10^{-8}$ m. The numerics are performed on the rescaled domain *B* and the homogeneous medium is taken to be vacuum ($\varepsilon _m=\varepsilon _0$ and $\mu _m=\mu _0$). The physical parameter values are summarised in Table 1.

**Table 1:** Physical constants and parameters values

Symbol	Magnitude
$\omega _p$	$2\times 10^{15}$ Hz
*T*	$10^{-14}$s
$\varepsilon _0$	$8.854187128 \times 10^{-12}$ $\hbox {Fm}^{-1}$
$\mu _0$	$4\pi \times 10^{-7}$ $\hbox {Hm}^{-1}$
$\delta$	$10^{-8}$ m
*d*	$(1/\sqrt{2},1/\sqrt{2})$
*z*	(0, 0)

### Modes contribution decay

**Fig. 2:**
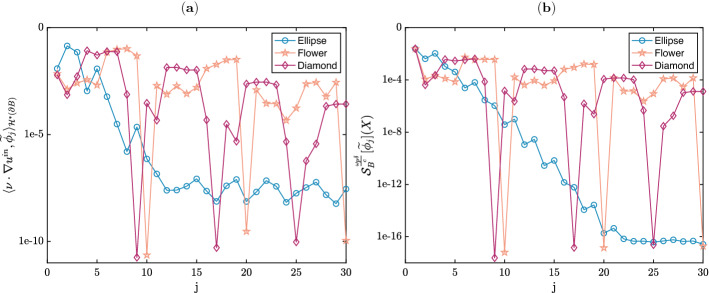
We illustrate on a logarithmic scale the fast decay of the modal expansion terms by plotting the scalar products $\langle \nu \cdot \nabla u^\text {in},{\widetilde{\phi }}_j\rangle _{{\mathcal {H}}^*(\partial B)}$ on panel (**a**) and the modes ${\mathcal {S}}^{\omega _p\delta c^{-1}}_B[{\widetilde{\phi }}_j](X)$ on panel (**b**), against *j*, for $1\le j\le 30$, for the diamond, the ellipse and the flower

It was shown in Sect. 3.1 that the scalar products $\langle {\widetilde{F}},{\widetilde{\phi }}_j\rangle _{{\mathcal {H}}^*(\partial B)}$ decay very rapidly when $d=3$. In a two-dimensional setting, the theoretical framework is not as clear, but we check numerically that the contribution the modes decrease quite fast with *j*. Recall that the weight of the $j^\text {th}$ mode is given by the scalar product $\langle {\widetilde{F}},{\widetilde{\phi }}_j\rangle _{{\mathcal {H}}^*(\partial B)}$, which, in a low-frequency regime, can be approximated as $\langle \nu \cdot \nabla u^\text {in},{\widetilde{\phi }}_j\rangle _{{\mathcal {H}}^*(\partial B)}$ (see Lemma 15). On panel (a) of Fig. 2 we show on all examples that $\langle \nu \cdot \nabla u^\text {in},{\widetilde{\phi }}_j\rangle _{{\mathcal {H}}^*(\partial B)}$ decays as *j* grows. We average over all possible directions *d* of the incident field. Panel (b) of the same picture shows that the modes themselves, ${\mathcal {S}}^{\omega _p\delta c^{-1}}_B[{\widetilde{\phi }}_j](X)$, decrease as *j* increases. We average here over all observation positions, *X* belongs to a circle of radius 100 centred at $z=0$.

### Plasmonic resonances

**Fig. 3:**
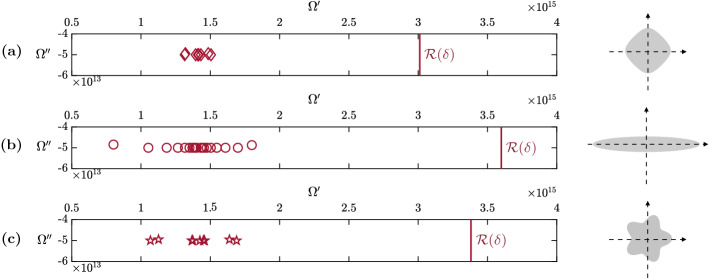
We plot, for the diamond (**a**), the ellipse (**b**) and the flower (**c**), the two-dimensional first-order corrected resonances with positive real parts from (16): $\varOmega ^+_j (\delta )= \varOmega _j' + i \varOmega _j''$, for $j=1,\ldots ,20$. These resonances lie in the lower part of the complex plane and their real part is between $\omega _p/4$ and $\omega _p$ (and smaller than ${\mathcal {R}}(\delta )$). Their negative counterparts are symmetric with respect to the imaginary axis

We plot the first-order corrected plasmonic resonances with positive real parts on Fig. 3. The resonance radius ${\mathcal {R}}(\delta )$ from definition 6 is drawn as a red vertical line on the three subplots and is shown to encompass all the low-frequency resonances.

We can then verify a posteriori that our choice of size $\delta$ is consistent by checking that ${\mathcal {R}}(\delta )$ is still in the *low-frequency* region, see Table 2.

**Table 2:** Validity check

*B*	${\mathcal {R}}(\delta )$	${\mathcal {R}}(\delta ) \delta c^{-1}$
Diamond	$3.0117e+15$	0.1005
Ellipse	$3.6228e+15$	0.1208
Flower	$3.3806e+15$	0.1128

### Validation of theorem 5

In this section, we validate the two-dimensional approximation of the scattered wave in the time domain given in Theorem 5 by plotting the asymptotic result against full numerical simulations.

**Fig. 4:**
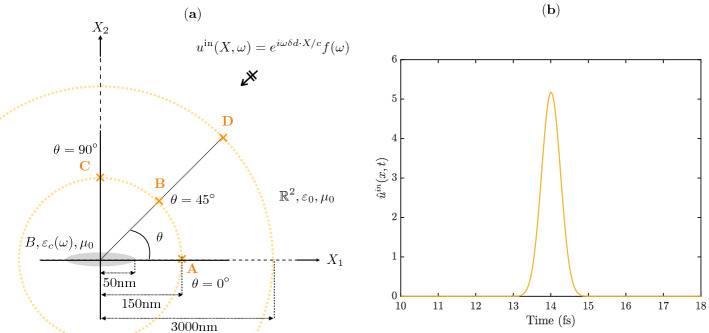
Not-to-scale sketch of the simulation setting for the ellipse on panel (**a**). The observation points *A*, *B* and *C* are placed on a circle of radius 150 nm ($|X|=15$) centred at the origin, while observation point *D* is placed in the far-field on a circle of radius 3000 nm ($|X|=300$) at angle $\theta _D=45^\circ$. On panel (**b**) we plot the time domain incident wave $\hat{u}^{\text {in}}(x,t)$ from (23) at $x=3000$ nm

We sketch the simulation setting with the ellipse in Fig. 4a. We define three observation points *A*, *B* and *C* on a circle of radius 150 nm ($|X|=15$) and one observation point *D* on a circle of radius 3000 nm ($|X|=300$). They are characterised by their angle with respect to the x-axis: $\theta _A=0^\circ$, $\theta _B=\theta _D=45^\circ$, $\theta _C=90^\circ$. The nanoparticle is illuminated by a plane wave of the form $u^\text {in}(X)=e^{i k_m d\cdot \delta X} f(\omega )$ where *f* is the Fourier transform of a bump function compactly supported in the interval $[0,C_1]$, with $C_1= 8$ fs. We plot the time domain incoming wave in Fig. 4b. To ease the notations we drop the tilde subscript in the following and write *u*(*X*) instead of ${\widetilde{u}}(X)$.

#### Reference solution

We call *reference solution* the low-frequency part of the scattered field in the time domain. We first uniformly discretise our frequency domain $I_\omega$ in $L=10^4$ points, with

$$\begin{aligned} I(\omega ):=\&\, [-\rho \delta c^{-1}, -\epsilon \delta c^{-1}] \cup [\epsilon \delta c^{-1}, \rho \delta c^{-1}] \\ =\&\, [-\omega _p \delta c^{-1}, -\omega _p \delta c^{-1}/4]\cup [\omega _p \delta c^{-1}/4,\omega _p \delta c^{-1}], \end{aligned}$$

by setting $\omega '_{l} \in I(\omega )$ such that:

$$\begin{aligned}&-\rho \delta c^{-1}=\omega '_{-L}< \omega '_{-L+1}< \cdots< \omega '_{-1}= -\epsilon \delta c^{-1}, \\&\quad \epsilon \delta c^{-1} =\omega '_{1}< \cdots< \omega '_{L-1} < \omega '_{L}=\rho \delta c^{-1}, \end{aligned}$$

with $\omega '_{l+1}-\omega '_{l}=(\rho -\epsilon )\delta c^{-1}/L$ for every $l \in [-L-1,-1]\cup [1,L-1]$. We compute the scattered field in the frequency domain using the representation formula (3). The single-layer potential is approximated using $N=2^8$ equally-spaced discretisation points along the boundary $\partial B$. We define the dimensionless frequency $\omega '=\omega \delta c^{-1}$. The reference solution is computed by taking the truncated inverse Fourier transform

25 $$\begin{aligned} P_{\rho ,\epsilon }\left[ {u}^\text {sca}\right] (X, t) \approx \frac{(\rho - \epsilon )}{L} \sum _{l=1}^L \left( e^{-i\omega '_{-l}c \delta ^{-1}t}u^\text {sca}\left( X,\omega '_{-l}c \delta ^{-1}\right) + e^{-i\omega '_{l}c \delta ^{-1}t}u^\text {sca}\left( X,\omega '_{l}c \delta ^{-1}\right) \right) . \end{aligned}$$

#### Asymptotic solution

The expansion is obtained by summing the first $J=30$ modes. Using Theorem 5, the modal approximation of order *J* becomes:

26 $$\begin{aligned} \begin{aligned} U_J(X,t)=&\,2 \pi i \sum _{j=1}^J\varepsilon _0 \left( \frac{\left( {\varOmega ^+_j(\delta )}^2 + i \varOmega ^+_j(\delta ) T^{-1} -\omega _p^2\right) \langle {\widetilde{F}}, {\widetilde{\phi }}_j \rangle _{{\mathcal {H}}^*(\partial B)}}{\left( 1+\left( \omega _p\delta c^{-1}\right) ^2 \log \left( \varOmega ^+_{s,j}\delta c^{-1}\right) \alpha _j\right) \left( \varOmega ^+_j(\delta )-\varOmega ^-_j(\delta )\right) } \right. \\&\, \left. \times \delta {\mathcal {S}}^{\varOmega ^+_j(\delta )\delta c^{-1}}_B[{\widetilde{\phi }}_j](X) e^{-i\varOmega ^+_j(\delta )t} \right. \\&\, \left. + \,\frac{\left( {\varOmega ^-_j(\delta )}^2 + i \varOmega ^-_j(\delta ) T^{-1} -\omega _p^2\right) \langle {\widetilde{F}}, {\widetilde{\phi }}_j \rangle _{{\mathcal {H}}^*(\partial B)}}{\left( 1+\left( \omega _p\delta c^{-1}\right) ^2 \log \left( \varOmega ^-_{s,j}\delta c^{-1}\right) \alpha _j\right) \left( \varOmega ^-_j(\delta )-\varOmega ^+_j(\delta )\right) } \right. \\&\,\left. \times \delta {\mathcal {S}}^{\varOmega ^-_j(\delta )\delta c^{-1}}_B[{\widetilde{\phi }}_j](X) e^{-i\varOmega ^-_j(\delta )t}\right) . \end{aligned} \end{aligned}$$

**Fig. 5:**
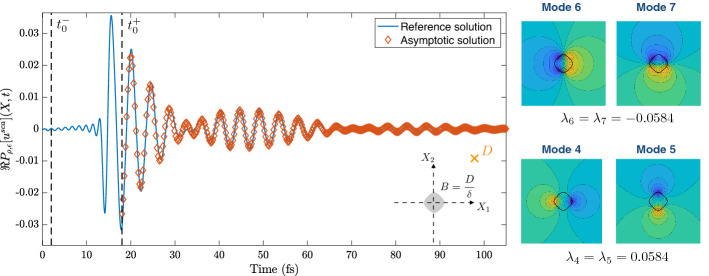
The real part of the reference solution (blue line) from (25) against the real part of the asymptotics (orange symbols) from (26) for the diamond, from observation point $X=D$. The four modes with the largest amplitude are shown on the right (order left to right, up to down)

**Fig. 6:**
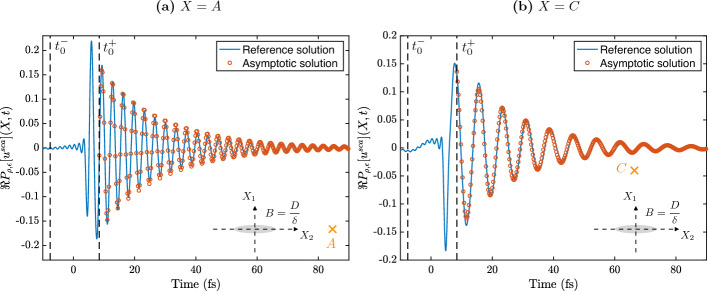
The real part of the reference solution (blue line) from (25) against the real part of the asymptotics (orange symbols) from (26) for the ellipse, from observation point $X=A$ on panel (**a**) and $X=C$ on panel (**b**)

**Fig. 7:**
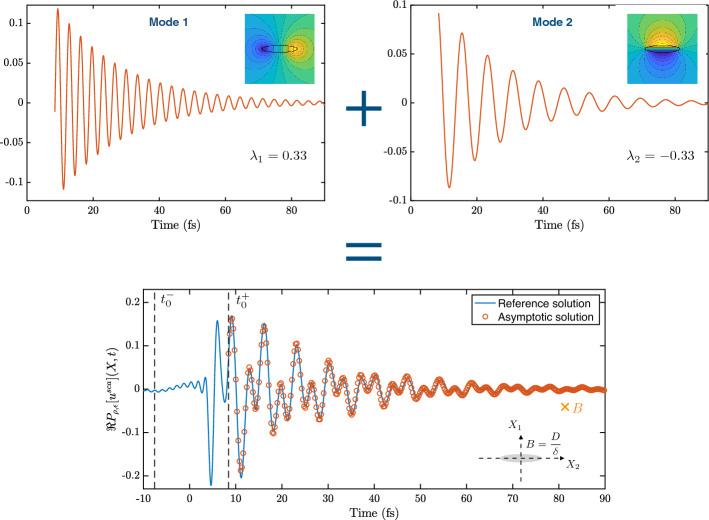
The real part of the field scattered by the ellipse and observed at point $X=B$ is the superposition of two dipoles modes. The modes (upper panels) oscillate at different frequencies. On the lower panel, the reference solution from (25) captures well the expansion from (26)

**Fig. 8:**
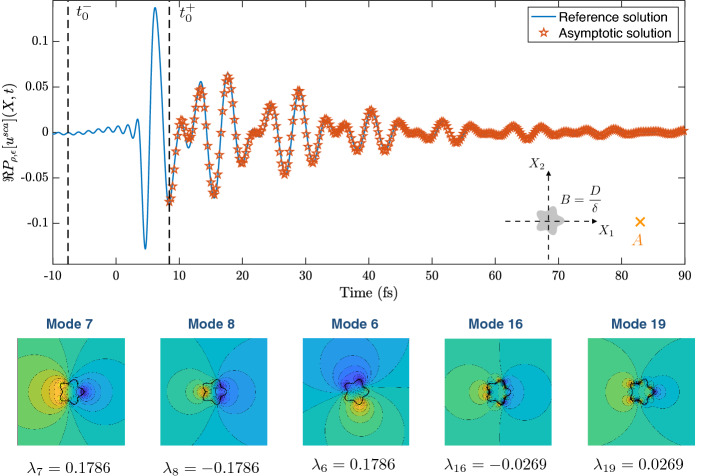
We plot the real part of the reference solution (25) as a blue line against the real part of the asymptotic one (26) as orange symbols for the flower. The observation point is at $X=A$ (shown on the not-to-scale inset). The five modes with the largest amplitude are shown on the bottom (order left to right)

The simulation results are shown in Figs. 5, 6, 7 and 8 . To corroborate our pole expansion, we plot the real part of the reference solution (25) against the real part of the asymptotic one (26) for the different domains and from different observation points.

#### Comparison in the far-field for the diamond

We begin with the diamond, since it is the shape that satisfies the hypotheses of Theorem 5. Figure 5 shows the field scattered by the diamond, measured in the far-field at position $X=D$. The reference solution is nicely approximated by the sum of four modes (4, 5, 6 and 7).

#### Extension to a nearer-field for the ellipse and flower

Figure 6 shows the field scattered by the ellipse, measured at position $X=A$ on panel (a) and $X=C$ on panel (b). In both cases the time domain scattered wave (blue line) is well approximated by the sum of decaying modes (orange symbols). Although we compute the first 30 terms of the modal expansion, the actual number of modes which contribute significantly to approximate the reference solution is much smaller. Indeed, only 1 mode is necessary to reconstruct more than $99\%$ of the signal in Fig. 6.

When the observation point is at $X=B$, we illustrate in Fig. 7 that two modes are needed to match the reference solution for the ellipse. Mode 1, corresponding to a dipole which radiates most of the energy along the x-axis, is associated to the eigenvalue $\lambda _1=0.33$. Mode 2 corresponds to the dipole which radiates most of the energy along the y-axis and is associated to the eigenvalue $\lambda _1=-0.33$. Mode 1 oscillates slightly faster than mode 2, resulting in the double oscillation visible on the lower plot. These numerical simulations are in line with [9]. Even relatively close to the particle (the observation distance is about a tenth of the wavelength), only two modes radiate in the far-field.

Figure 8 shows that even for the non-algebraic flower shape, the scattered wave (blue line) is well approximated by the sum of a small number of decaying modes (orange symbols). As anticipated by Fig. 2, the modes decay being faster for the ellipse than it is for the flower, a larger number of modes is needed for the latter. In Fig. 8, eight modes were needed to reconstruct more than $99\%$ of the reference solution (and five modes sufficed for $95\%$).

#### About the high frequencies

**Fig. 9:**
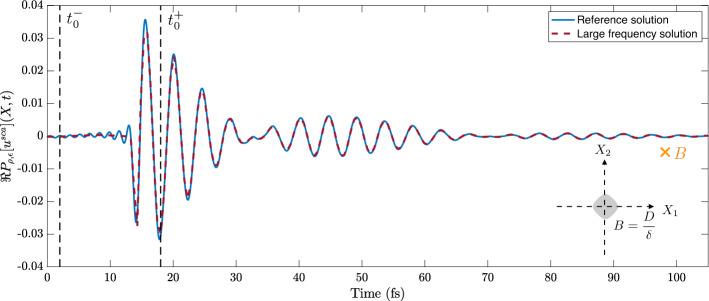
Reference (low-frequency) solution (computed with $\rho ={\mathcal {R}}(\delta )$) against large-frequency solution (computed with $\rho =100{\mathcal {R}}(\delta )$)

On Fig. 9 we show that the low-frequency part of the time domain solution is actually a good approximation of the full solution, as mentioned in Remark 11. It is completely non-trivial, as we have no information on the localisation of poles for the resolvent in the frequency domain outside the *low-frequency* range. It seems that there are no more resonances in the high-frequency range due to the dispersive nature of the material. This will be investigated in future work.

#### About the computational cost

We note that, because a small number of modes usually suffices to approximate the reference solution, the computation cost of the asymptotic solution is relatively cheap. The time needed to compute the reference solution and the asymptotic one are linear in $L(= 10^4)$ and $J(= 30)$, respectively. Thus, the time to compute the asymptotic solution is much smaller than the time to compute the reference solution, namely, a hundred times smaller. Moreover, the modes can be pre-calculated and one can compute at a low cost the response of the particle to any given illumination in the time domain.

## Concluding remarks

In this paper, we have shown that it is possible to define quasi-normal modes (similar to the ones found in the physics literature) for *small* plasmonic particles using the spectral decomposition of the Neumann–Poincaré operator and some perturbative spectral analysis. We have proved that, in a three-dimensional setting, only a few modes are necessary to represent the solutions of the scattering problem by a strictly convex plasmonic particle and that these types of representations can give a very good approximation of the field in the time domain. Our numerical simulations have corroborated the validity of this approach in the two-dimensional case. This theoretical and numerical framework can be adapted to handle more complex systems with multiple particles (see [6]). This work needs to be extended to solutions of Maxwell’s equations and to dielectric structures. This will be the subject of forthcoming papers.

## Properties of the layer potentials

We briefly recall here some basic properties of layer potential theory. There is abundant literature on the subject. For more details we refer to the books [3, 4, 18, 35].

### Definitions and notations

#### Definition 7

Denote by $\varGamma ^k$ the outgoing Green’s function for the homogeneous medium, i.e., the unique solution of the Helmholtz operator:

$$\begin{aligned} \left( \varDelta +k^2\right) \varGamma ^k(\cdot ,y)=\delta _y(\cdot ) \quad \text { in }{\mathbb {R}}^d, \end{aligned}$$

satisfying the Sommerfeld radiation condition. In three dimensions, $\varGamma ^k$ is given by

$$\begin{aligned} \varGamma ^k(x,y)=-\frac{e^{ik|x-y|}}{4\pi |x-y|}, \qquad x,y \in {\mathbb {R}}^3. \end{aligned}$$

In two dimensions, it is given by

$$\begin{aligned} \varGamma ^{k}(x,y)= {\left\{ \begin{array}{ll} \frac{1}{2\pi }\log |x-y|, &{}\text{ if } k=0, \\ -\frac{i}{4}H_0^{(1)}{(k|x-y|)}, &{}\text{ if } k>0, \end{array}\right. } \end{aligned}$$

for $x,y \in {\mathbb {R}}^2$, where $H_0^{(1)}$ is the well-known Hankel function of the first kind and order 0.

#### Lemma 5

The Hessian matrix of the outgoing fundamental solution in three dimensions ${\mathbf {D}}_x^2 \varGamma ^k (x,z)=(D)_{p,q=1}^3$ is with entries

$$\begin{aligned} D_{pp}&=\frac{e^{ik|x-z|}}{4\pi |x-z|^5}\left[ |x-z|^2-3(x_p-z_p)^2+3ik(x_p-z_p)^2|x-z|+k^2 |x-z|^2-ik |x-z|^3\right] , \\ D_{pq}&=\frac{e^{ik|x-z|}}{4\pi |x-z|^5}(x_p-z_p)(x_q-z_q)\left[ -3+3ik|x-z|+k^2|x-z|^2\right] , \qquad \text {for~} p\ne q. \end{aligned}$$

#### Definition 8

For a function $\phi \in L^2(\partial D)$, we define the single-layer potential by

$$\begin{aligned} {\mathcal {S}}^{k}_D[\phi ](x)=\int _{\partial D} \varGamma ^k(x,y)\phi (y)\mathrm {d}\sigma (y), \qquad x \in {\mathbb {R}}^d, \end{aligned}$$

and the Neumann–Poincaré operator by

$$\begin{aligned} {\mathcal {K}}^{k,*}_D[\phi ](x)=\int _{\partial D} \frac{\partial \varGamma ^k(x,y)}{\partial \nu (x)}\phi (y)\mathrm {d}\sigma (y), \qquad x \in \partial D. \end{aligned}$$

When $k=0$, we just write ${\mathcal {S}}_D$ and ${\mathcal {K}}_D^*$ for simplicity.

### The Calderón identity and symmetrisation of ${\mathcal {K}}_D^*$

#### Lemma 6

We recall the following classical results [10, 24, 35].

1. The following Plemelj’s symmetrisation principle identity (also known as Calderón) holds:
  27 $$\begin{aligned} {\mathcal {K}}_D {\mathcal {S}}_D = {\mathcal {S}}_D {\mathcal {K}}_D^* \qquad \text{ on } H^{-\frac{1}{2}}(\partial D). \end{aligned}$$
2. If $\partial D\in C^{1,\alpha }$, for some $\alpha >0$, then ${\mathcal {K}}_D^*$ is compact. Let $(\lambda _j,\varphi _j)_{j\in {\mathbb {N}}}$ be the eigenvalues and normalised eigenfunctions of ${\mathcal {K}}_D^*$ in ${\mathcal {H}}^*(\partial D)$. Then $\lambda _j \in ]-1/2, 1/2]$, $\lambda _0=1/2$ and $\lambda _j \rightarrow 0$ as $j\rightarrow \infty$.
3. The operator ${\mathcal {K}}_D^*$ is self-adjoint in the Hilbert space ${\mathcal {H}}^*(\partial D)$ which is $H^{-\frac{1}{2}}(\partial D)$ equipped with the following inner product:
  $$\begin{aligned} \left\langle u, v\right\rangle _{{\mathcal {H}}^*(\partial D)} = {\left\{ \begin{array}{ll} \displaystyle -\langle u, \widetilde{{\mathcal {S}}}_D[v]\rangle _{-\frac{1}{2},\frac{1}{2}}, &{} d=2, \\ \displaystyle -\left\langle u, {\mathcal {S}}_D[v]\right\rangle _{-\frac{1}{2},\frac{1}{2}}, &{} d=3, \end{array}\right. } \end{aligned}$$
  where
  $$\begin{aligned} \widetilde{{\mathcal {S}}}_D[v] = {\left\{ \begin{array}{ll} {\mathcal {S}}_D[v] &{} \text {if } \langle v, \chi (\partial D)\rangle _{-\frac{1}{2}, \frac{1}{2}} = 0,\\ -\chi (\partial D) &{} \text {if } v = \varphi _0, \end{array}\right. } \end{aligned}$$
  with $\varphi _0$ being the unique (in the case of a single particle) eigenfunction of ${\mathcal {K}}^*_D$ associated with eigenvalue 1/2 such that $\langle \varphi _0, \chi (\partial D)\rangle _{-\frac{1}{2}, \frac{1}{2}}=1$. Also, $\left\langle \cdot ,\cdot \right\rangle _{-\frac{1}{2},\frac{1}{2}}$ is the duality pairing between $H^{-\frac{1}{2}}(\partial D)$ and $H^{\frac{1}{2}}(\partial D)$.
4. From [10], we have the following extension of (27) in two dimensions:
  $$\begin{aligned} {\mathcal {K}}_D \widetilde{{\mathcal {S}}}_D = \widetilde{{\mathcal {S}}}_D {\mathcal {K}}_D^* \qquad \text{ on } H^{-\frac{1}{2}}(\partial D). \end{aligned}$$
5. Since ${\mathcal {K}}_D\left[ \chi \left( \partial D\right) \right] =\dfrac{1}{2} \chi (\partial D)$, it holds that
  $$\begin{aligned} \int _{\partial D} \varphi _j =0 \qquad \text{ for } j\ne 0. \end{aligned}$$
6. The following trace formulae hold for $\phi \in H^{-\frac{1}{2}}(\partial D)$:
  $$\begin{aligned} {\mathcal {S}}^k_D[\phi ]|_+= & {} {\mathcal {S}}^k_D[\phi ]|_- ,\\ \left. \frac{\partial {\mathcal {S}}^k_D[\phi ]}{\partial \nu }\right| _{\pm }= & {} \left( \pm \frac{1}{2}I+{\mathcal {K}}^{k,*}_D\right) [\phi ], \end{aligned}$$
  where *I* denotes the identity operator.
7. The following representation formula holds:
  $$\begin{aligned} {\mathcal {K}}_D^*[\phi ]=\sum _{j=0}^\infty \lambda _j \left\langle \phi ,\varphi _j\right\rangle _{{\mathcal {H}}^*(\partial D)} \varphi _j, \qquad \forall \phi \in {\mathcal {H}}^*(\partial D). \end{aligned}$$

### Invertibility of the boundary operators

#### Lemma 7

For *k* small enough, the three-dimensional single-layer potential ${\mathcal {S}}^k_D:H^{-1/2}(\partial D)\rightarrow H^{1/2}(\partial D)$ is invertible.

#### Lemma 8

${\mathcal {S}}_D:H^{-1/2}(\partial D)\rightarrow H^{1/2}(\partial D)$ is invertible in three dimensions.

In two dimensions, the single-layer potential ${\mathcal {S}}_D:H^{-1/2}(\partial D)\rightarrow H^{1/2}(\partial D)$ is, in general, not invertible. All the proofs for the following lemmas can be found in [6].

#### Lemma 9

For *k* small enough, the two-dimensional boundary operator $\widehat{{\mathcal {S}}}_D^k:{\mathcal {H}}^*(\partial D)\rightarrow {\mathcal {H}}^*(\partial D)$ defined as

28 $$\begin{aligned} \widehat{{\mathcal {S}}}_D^{k}[\phi ](x)={\mathcal {S}}_D^0[\phi ](x)+\eta _k\int _{\partial D}\phi (y)\mathrm {d}\sigma (y), \end{aligned}$$

is invertible and

29 $$\begin{aligned} \left( \widehat{{\mathcal {S}}}_D^k\right) ^{-1}=\widetilde{{\mathcal {S}}}_D^{-1}-\left\langle \widetilde{{\mathcal {S}}}_D^{-1}[\cdot ],\varphi _0\right\rangle _{{\mathcal {H}}^*(\partial D) }\varphi _0-{\mathcal {U}}_k, \end{aligned}$$

where ${\mathcal {U}}_k=\dfrac{\left\langle \widetilde{{\mathcal {S}}}_D^{-1}[\cdot ],\varphi _0\right\rangle _{{\mathcal {H}}^*(\partial D)}}{{\mathcal {S}}_D[\varphi _0]+\eta _k} \varphi _0$ and $\eta _k=(1/2\pi )(\log {k}+\gamma -\log {2})-i/4,$ with the constant $\gamma$ being the Euler constant. Note that ${\mathcal {U}}_k={\mathcal {O}}(1/ \log k)$.

#### Lemma 10

For *k* small enough, the two-dimensional single-layer potential ${\mathcal {S}}_D^k:{\mathcal {H}}^*(\partial D)\rightarrow {\mathcal {H}}^*(\partial D)$ is invertible.

## Scaling properties for a finite volume particle

For each function *f* defined on $\partial D$, we define a corresponding function on $\partial B$ by ${\widetilde{f}}(X):=f(z+\delta X)$.

### Lemma 11

It holds that

$$\begin{aligned} {\mathcal {K}}^{k,*}_D[f](x)= & {} {\mathcal {K}}^{k\delta ,*}_B[{\widetilde{f}}](X),\\ {\mathcal {S}}^{k}_D[f](x)= & {} \delta {\mathcal {S}}^{k\delta }_B[{\widetilde{f}}](X). \end{aligned}$$

### Lemma 12

For *f*, *g* defined on $\partial D$, corresponding to ${\widetilde{f}},{\widetilde{g}}$, respectively, we have

$$\begin{aligned} \left\langle f,g \right\rangle _{{\mathcal {H}}^*(\partial D)}= & {} {\left\{ \begin{array}{ll} \delta ^2\left\langle {\widetilde{f}},{\widetilde{g}} \right\rangle _{{\mathcal {H}}^*(\partial B)}, &{} d=2,\\ \delta ^3\left\langle {\widetilde{f}},{\widetilde{g}} \right\rangle _{{\mathcal {H}}^*(\partial B)}, &{} d=3, \end{array}\right. } \\ ||f||_{{\mathcal {H}}^*(\partial D)}= & {} {\left\{ \begin{array}{ll} \delta ||{\widetilde{f}}||_{{\mathcal {H}}^*(\partial B)}, &{} d=2, \\ \delta ^{3/2}||{\widetilde{f}}||_{{\mathcal {H}}^*(\partial B)}, &{} d=3. \end{array}\right. } \end{aligned}$$

### Proof

In three dimensions, by straightforward calculations we have

$$\begin{aligned} \begin{aligned} \left\langle f,g \right\rangle _{{\mathcal {H}}^*(\partial D)}&= \int _{\partial D} f(x) \int _{\partial D} \frac{g(y)}{4\pi |x-y|} \mathrm {d}\sigma (y) \mathrm {d}\sigma (x) \\&= \delta ^3 \int _{\partial B} f(z+\delta X) \int _{\partial B} \frac{g(z+\delta Y)}{4 \pi |X-Y|} \mathrm {d}\sigma (Y) \mathrm {d}\sigma (X) \\&= \delta ^3 \left\langle {\widetilde{f}},{\widetilde{g}} \right\rangle _{{\mathcal {H}}^*(\partial B)}. \end{aligned} \end{aligned}$$

Hence, $$||f||_{{\mathcal {H}}^*(\partial D)} = \delta ^{3/2}||{\widetilde{f}}||_{{\mathcal {H}}^*(\partial B)}$$.

In the two-dimensional case we write ${\mathcal {H}}^*(\partial D) = {\mathcal {H}}_0^*(\partial D) \oplus \{\mu \varphi _0, \; \mu \in {\mathbb {C}}\}$ and treat both cases: *g* belongs to either ${\mathcal {H}}_0^*(\partial D)$ or $\{\mu \varphi _0, \; \mu \in {\mathbb {C}}\}$. In the former case, we have

$$\begin{aligned} \begin{aligned} \left\langle f,g \right\rangle _{{\mathcal {H}}^*(\partial D)}&= -\frac{1}{2\pi }\int _{\partial D} f(x) \int _{\partial D} g(y) \log (|x-y|) \mathrm {d}\sigma (y) \mathrm {d}\sigma (x) \\&= -\frac{\delta ^2}{2\pi } \int _{\partial B} f(z+\delta X) \int _{\partial B} g(z+\delta Y) (\log (\delta ) + \log (|X-Y|)) \mathrm {d}\sigma (Y) \mathrm {d}\sigma (X) \\&= \delta ^2 \left\langle {\widetilde{f}},{\widetilde{g}} \right\rangle _{{\mathcal {H}}^*(\partial B)}. \end{aligned} \end{aligned}$$

If $g = \mu \varphi _0$, we have

$$\begin{aligned} \begin{aligned} \left\langle f,g \right\rangle _{{\mathcal {H}}^*(\partial D)}&= \int _{\partial D} \mu f(x) \mathrm {d}\sigma (x) \\&= \delta \int _{\partial B} \mu f(z+\delta X) \mathrm {d}\sigma (X) \\&= \delta ^2 \left\langle {\widetilde{f}},{\widetilde{g}} \right\rangle _{{\mathcal {H}}^*(\partial B)}, \end{aligned} \end{aligned}$$

where the last equality follows from the fact that $\delta {\widetilde{\varphi }}_0$ is the (unique) eigenfunction of ${\mathcal {K}}^*_B$ associated with eigenvalue 1/2 such that $\langle \delta {\widetilde{\varphi }}_0, \chi (\partial B) \rangle _{-\frac{1}{2}, \frac{1}{2}}=1$. Hence, $||f||_{{\mathcal {H}}^*(\partial D)} = \delta ||{\widetilde{f}}||_{{\mathcal {H}}^*(\partial B)}$. $\square$

## Asymptotic expansions

### Asymptotic expansions of the boundary operators

#### Lemma 13

1. The three-dimensional single-layer potential and its inverse admit the following expansions in the quasi–static limit $k\delta \rightarrow 0$:
  $$\begin{aligned} {\mathcal {S}}^{k\delta }_B= & {} {\mathcal {S}}_B+k\delta {\mathcal {S}}_{B,1}+\left( k\delta \right) ^2{\mathcal {S}}_{B,2}+{\mathcal {O}}\left( \left( k\delta \right) ^3\right) , \\ \left( {\mathcal {S}}^{k\delta }_B\right) ^{-1}= & {} {\mathcal {S}}^{-1}_B+k\delta {\mathcal {B}}_{B,1}+\left( k\delta \right) ^2{\mathcal {B}}_{B,2}+{\mathcal {O}}\left( \left( k\delta \right) ^3\right) , \end{aligned}$$
  where, for $$\phi \in H^{-\frac{1}{2}}(\partial B)$$,
  $$\begin{aligned} {\mathcal {S}}_{B,j}[\phi ](x)=-\frac{i}{4\pi }\int _{\partial B} \frac{(i|x-y|)^{j-1}}{j!}\phi (y)\mathrm {d}\sigma (y), \qquad x \in {\mathbb {R}}^3, \end{aligned}$$
  for $j\in {\mathbb {N}}$. Also ${\mathcal {B}}_{B,1}=-{\mathcal {S}}^{-1}_B{\mathcal {S}}_{B,1}{\mathcal {S}}^{-1}_B$ and ${\mathcal {B}}_{B,2}=-{\mathcal {S}}^{-1}_B{\mathcal {S}}_{B,2}{\mathcal {S}}^{-1}_B+{\mathcal {S}}^{-1}_B{\mathcal {S}}_{B,1}{\mathcal {S}}^{-1}_B{\mathcal {S}}_{B,1}{\mathcal {S}}^{-1}_B$.
2. The two-dimensional single-layer potential and its inverse admit the following expansions in the quasi–static limit $k\delta \rightarrow 0$:
  $$\begin{aligned} {\mathcal {S}}^{k\delta }_B= & {} \widehat{{\mathcal {S}}}_B^{k\delta }+(k\delta )^2\log (k\delta ) {\mathcal {S}}_{B,1}^{(1)} +{\mathcal {O}}\left( \left( k\delta \right) ^2\right) , \\ \left( {\mathcal {S}}^{k\delta }_B\right) ^{-1}= & {} {\mathcal {L}}_B+{\mathcal {U}}_{k\delta } - (k\delta )^2\log (k\delta ) {\mathcal {L}}_B{\mathcal {S}}_{B,1}^{(1)} {\mathcal {L}}_B +{\mathcal {O}}\left( \left( k\delta \right) ^2\right) , \end{aligned}$$
  where, for $$\phi \in H^{-\frac{1}{2}}(\partial B)$$,
  $$\begin{aligned} {\mathcal {S}}_{B,1}^{(1)}[\phi ](x)= & {} -\frac{1}{8\pi } \int _{\partial B}|x-y|^{2}\phi (y)\mathrm {d}\sigma (y), \qquad x \in {\mathbb {R}}^2.\\ \end{aligned}$$
  Also ${\mathcal {P}}_{{\mathcal {H}}_0^*}$ is the orthogonal projection onto ${\mathcal {H}}_0^*$, ${\mathcal {L}}_B = {\mathcal {P}}_{{\mathcal {H}}_0^*}\widetilde{{\mathcal {S}}}^{-1}_B$.
3. The Neumann–Poincaré operator in three dimensions admits the following expansion in the quasi–static limit
  $$\begin{aligned} {\mathcal {K}}^{k\delta ,*}_B={\mathcal {K}}^*_B+\left( k\delta \right) ^2{\mathcal {K}}^{*}_{B,2}+{\mathcal {O}}\left( \left( k\delta \right) ^3\right) , \end{aligned}$$
  where, for $$\phi \in H^{-\frac{1}{2}}(\partial B)$$,
  $$\begin{aligned} {\mathcal {K}}^{*}_{B,2}[\phi ](x)=\frac{1}{8\pi }\int _{\partial B}\frac{(x-y)\cdot \nu (x)}{|x-y|}\phi (y)\mathrm {d}\sigma (y), \qquad x \in \partial B. \end{aligned}$$
4. The Neumann–Poincaré operator in two dimensions admits the following expansion in the quasi–static limit
  $$\begin{aligned} {\mathcal {K}}^{k\delta ,*}_B={\mathcal {K}}^*_B+ (k\delta )^2 \log (k\delta ) {\mathcal {K}}^{(1)}_{B,1} +{\mathcal {O}}\left( \left( k\delta \right) ^2\right) , \end{aligned}$$
  where, for $$\phi \in H^{-\frac{1}{2}}(\partial B)$$,
  $$\begin{aligned} {\mathcal {K}}^{(1)}_{B,1}[\phi ](x)=-\frac{1}{8\pi } \int _{\partial B} \frac{\partial |x-y|^{2}}{\partial \nu _x}\phi (y)\mathrm {d}\sigma (y), \qquad x \in \partial B. \end{aligned}$$

#### Proof

The proof can be found in [6]. $\square$

### Proof of lemma 1

#### Proof

Recall that

$$\begin{aligned} {\mathcal {A}}_B^{\omega \delta c^{-1}}=\frac{1}{\varepsilon _m}\left( \frac{1}{2}I+{\mathcal {K}}^{k_m\delta ,*}_B\right) +\frac{1}{\varepsilon _c}\left( \frac{1}{2}I-{\mathcal {K}}^{k_c\delta ,*}_B\right) \left( {\mathcal {S}}^{k_c\delta }_B\right) ^{-1}{\mathcal {S}}^{k_m\delta }_B. \end{aligned}$$

For the three-dimensional case, using Lemma 13 we have by straightforward calculations

$$\begin{aligned} {\mathcal {A}}^{\omega \delta c^{-1}}= & {} \frac{1}{\varepsilon _m}\left[ \frac{1}{2}I+{\mathcal {K}}_B^*+(k_m\delta )^2{\mathcal {K}}_{B,2}\right] +\frac{1}{\varepsilon _c}\left[ \frac{1}{2}I-{\mathcal {K}}_B^*-(k_c\delta )^2{\mathcal {K}}_{B,2}\right] \\&\times \left[ {\mathcal {S}}_B^{-1}+(k_c\delta ){\mathcal {B}}_{B,1}+(k_c\delta )^2{\mathcal {B}}_{B,2}\right] \\&\times \left[ {\mathcal {S}}_B+(k_m\delta ){\mathcal {S}}_{B,1}+(k_m\delta )^2{\mathcal {S}}_{B,2}\right] +{\mathcal {O}}\left( \left( \omega \delta c^{-1}\right) ^3\right) \\= & {} \frac{1}{2}\left( \frac{1}{\varepsilon _c}+\frac{1}{\varepsilon _m}\right) I-\left( \frac{1}{\varepsilon _c}-\frac{1}{\varepsilon _m}\right) {\mathcal {K}}^*_B+\left( \frac{(k_m\delta )^2}{\varepsilon _m}-\frac{(k_c\delta )^2}{\varepsilon _c}\right) {\mathcal {K}}_{B,2} \\&+\frac{1}{\varepsilon _c}\left( \frac{1}{2}-{\mathcal {K}}_B^*\right) \bigg ((k_m\delta ){\mathcal {S}}_B^{-1}{\mathcal {S}}_{B,1}+(k_m\delta )^2{\mathcal {S}}_B^{-1}{\mathcal {S}}_{B,2}\\&-k_c\delta {\mathcal {S}}_B^{-1}{\mathcal {S}}_{B,1}-k_c k_m\delta ^2{\mathcal {S}}_B^{-1}{\mathcal {S}}_{B,1}{\mathcal {S}}_B^{-1}{\mathcal {S}}_{B,1} +(k_c\delta )^2{\mathcal {S}}_B^{-1}{\mathcal {S}}_{B,1}{\mathcal {S}}_B^{-1}{\mathcal {S}}_{B,1},\\&-(k_c\delta )^2{\mathcal {S}}_B^{-1}{\mathcal {S}}_{B,2}\bigg )+{\mathcal {O}}\left( \left( \omega \delta c^{-1}\right) ^3\right) \\= & {} {\mathcal {A}}_B^0+\frac{1}{\varepsilon _c}\left( \frac{1}{2}I-{\mathcal {K}}_B^*\right) (k_m^2-k_c^2)\delta ^2{\mathcal {S}}_B^{-1}{\mathcal {S}}_{B,2} +{\mathcal {O}}\left( \left( \omega \delta c^{-1}\right) ^3\right) \\= & {} {\mathcal {A}}_B^0+\left( \omega \delta c^{-1}\right) ^2\frac{\varepsilon _m-\varepsilon _c}{\varepsilon _m\varepsilon _c}\left( \frac{1}{2}I-{\mathcal {K}}_B^*\right) {\mathcal {S}}_B^{-1}{\mathcal {S}}_{B,2} +{\mathcal {O}}\left( \left( \omega \delta c^{-1}\right) ^3\right) \end{aligned}$$

where we used ${\mathcal {S}}_B^{-1}{\mathcal {S}}_B=I$ and

$$\begin{aligned} \left( \frac{1}{2}I-{\mathcal {K}}_B^*\right) {\mathcal {S}}_B^{-1}{\mathcal {S}}_{B,1}=0. \end{aligned}$$

For the two-dimensional case, we have

$$\begin{aligned} \frac{1}{2}I - {\mathcal {K}}^{k_c \delta ,*}_B= & {} \frac{1}{2}I - {\mathcal {K}}_B^* - (k_c \delta )^2 \log (k_c \delta ) {\mathcal {K}}_{B,1}^{(1)} + {\mathcal {O}}\left( (k_c\delta )^2\right) ,\\ \left( {\mathcal {S}}^{k_c \delta }_B\right) ^{-1}= & {} {\mathcal {L}}_B + {\mathcal {U}}_{k_c \delta } - \left( \omega \delta c^{-1}\right) ^2 \log \left( \omega \delta c^{-1}\right) \frac{\varepsilon _c}{\varepsilon _m} {\mathcal {L}}_B {\mathcal {S}}^{(1)}_{B,1} {\mathcal {L}}_B + {\mathcal {O}}\left( \left( \omega \delta c^{-1}\right) ^2\right) ,\\ {\mathcal {S}}^{k_m \delta }_B= & {} {\widetilde{S}}_B + \varUpsilon _{k_m\delta } +\left( \omega \delta c^{-1}\right) ^2 \log \left( \omega \delta c^{-1}\right) {\mathcal {S}}^{(1)}_{B,1} + {\mathcal {O}}\left( \left( \omega \delta c^{-1}\right) ^2\right) . \end{aligned}$$

Also, $${\mathcal {L}}_B \varUpsilon _{k_m\delta } = {\mathcal {P}}_{{\mathcal {H}}^*_0}\widetilde{{\mathcal {S}}}_B^{-1} \varUpsilon _{k_m\delta } =0$$, where

$$\begin{aligned} \varUpsilon _{k_m\delta }[\psi ] = \langle \psi , {\widetilde{\phi }}_0 \rangle _{{\mathcal {H}}^*} ({\mathcal {S}}_B[{\widetilde{\phi }}_0] +\chi (\partial B) + \eta _{k_m \delta }). \end{aligned}$$

Hence,

$$\begin{aligned} ({\mathcal {S}}_B^{k_c\delta })^{-1}{\mathcal {S}}_B^{k_m\delta }= & {} {\mathcal {P}}_{{\mathcal {H}}^*_0}+{\mathcal {U}}_{k_c\delta } \widetilde{{\mathcal {S}}}_B + {\mathcal {U}}_{k_c\delta } \varUpsilon _{k_m \delta } + \left( \omega \delta c^{-1}\right) ^2 \log \left( \omega \delta c^{-1}\right) {\mathcal {L}}_B {\mathcal {S}}^{(1)}_{D,1} \left( I - \frac{\varepsilon _c}{\varepsilon _m} {\mathcal {P}}_{{\mathcal {H}}^*_0}\right) \\&+ {\mathcal {O}}\left( \left( \omega \delta c^{-1}\right) ^2\right) . \end{aligned}$$

We have that

$$\begin{aligned} \left( \frac{1}{2}I-{\mathcal {K}}_B^*\right) {\mathcal {U}}_{k_c\delta } = \dfrac{\left\langle \widetilde{{\mathcal {S}}}_B^{-1}[\cdot ],{\widetilde{\phi }}_0\right\rangle _{{\mathcal {H}}^*(\partial B)}}{{\mathcal {S}}_B[{\widetilde{\phi }}_0]+\eta _{k_c}} \left( \frac{1}{2}{\widetilde{\phi }}_0 - {\mathcal {K}}_B^*[{\widetilde{\phi }}_0]\right) = 0. \end{aligned}$$

$$\square $$

### Proof of Lemma 4

#### Proof

Using the Taylor expansion

$$\begin{aligned} \left. \varGamma ^{k_m}(z+\delta X,y)\right| _{X\in \partial B}=\varGamma ^{k_m}(z,y)+\delta X\cdot \nabla \varGamma ^{k_m}(z,y)+\delta ^2 X^\top {\mathbf {D}}_X^2 \varGamma ^{k_m} (z,y)X+\cdots , \end{aligned}$$

we compute, for $X\in \partial B$,

$$\begin{aligned} {\widetilde{F}}(X)= & {} {\widetilde{F}}_2(X)+\frac{1}{\delta \varepsilon _c}\left( \frac{1}{2}I-{\mathcal {K}}^{k_c\delta ,*}_B\right) \left( {\mathcal {S}}^{k_c\delta }_B\right) ^{-1}[{\widetilde{F}}_1](X) \\= & {} -\frac{1}{\delta \varepsilon _m}\frac{\partial \varGamma ^{k_m}(z+\delta X,y)}{\partial \nu _X}-\frac{1}{\delta \varepsilon _c}\left( \frac{1}{2}I-{\mathcal {K}}^*_B+{\mathcal {O}}\left( (k_c\delta )^2\right) \right) \\&\times \left( {\mathcal {S}}^{-1}_B+k_c\delta {\mathcal {B}}_{B,1}+{\mathcal {O}}\left( (k_c\delta )^2\right) \right) \left[ \varGamma ^{k_m}\left( z+\delta X,y\right) \right] \\= & {} -\frac{1}{\delta \varepsilon _m}\nu _X\cdot \nabla \varGamma ^{k_m}(z+\delta X,y)-\frac{1}{\delta \varepsilon _c}\left( \frac{1}{2}I-{\mathcal {K}}^*_B\right) {\mathcal {S}}^{-1}_B\left[ \varGamma ^{k_m}(z+\delta X,y)\right] \\&-\frac{k_c}{\varepsilon _c}\left( \frac{1}{2}I-{\mathcal {K}}^*_B\right) {\mathcal {B}}_{B,1}\left[ \varGamma ^{k_m}(z+\delta X,y)\right] +{\mathcal {O}}\left( \omega ^2 \delta c ^{-2}\right) \\= & {} - \frac{1}{\varepsilon _m}\nu _X\cdot \nabla \varGamma ^{k_m}(z,y)-\frac{\varGamma ^{k_m}(z,y)}{\delta \varepsilon _c}\left( \frac{1}{2}I-{\mathcal {K}}^*_B\right) {\mathcal {S}}^{-1}_B\left[ \chi (\partial B)\right] \\&-\frac{\nabla \varGamma ^{k_m}(z,y)}{\varepsilon _c}\cdot \left( \frac{1}{2}I-{\mathcal {K}}^*_B\right) {\mathcal {S}}^{-1}_B[X]+{\mathcal {O}}\left( \omega ^2 \delta c ^{-2}\right) \\= & {} -\frac{1}{\varepsilon _m}\nu _X\cdot \nabla \varGamma ^{k_m}(z,y)+\frac{1}{\varepsilon _c}\nu _X\cdot \nabla \varGamma ^{k_m}(z,y)+{\mathcal {O}}\left( \omega ^2 \delta c ^{-2}\right) , \end{aligned}$$

where we used $\left( \frac{1}{2}I-{\mathcal {K}}^*_B\right) {\mathcal {S}}^{-1}_B[\chi (\partial B)]=0$ and $\left( \frac{1}{2}I-{\mathcal {K}}^*_B\right) {\mathcal {B}}_{B,1}=0$. It is immediate to see that $\left( \frac{1}{2}I-{\mathcal {K}}^*_B\right) {\mathcal {S}}^{-1}_B[X]=-\nu _X$, indeed assuming there exists $\phi \in {\mathcal {H}}^*(\partial B)$ such that ${\mathcal {S}}^{-1}_B[X]=\phi$, then ${\mathcal {S}}_B[\phi ]=X$, and $\left. \partial {\mathcal {S}}_B[\phi ]/\partial \nu _X \right| _-= \nu _X$ which is equivalent to $\left( \frac{1}{2}I-{\mathcal {K}}^*_B\right) [\phi ]=-\nu _X$ using jump conditions. $\square$

### Proof of Lemma 15

#### Proof

Using the Taylor expansion

$$\begin{aligned} e^{ik_m d\cdot (z + \delta X)} = e^{ik_m d\cdot z} + \frac{i \omega }{c} [d \cdot \delta X]e^{ik_m d\cdot z} + {\mathcal {O}}\left( \left( k_m\delta \right) ^2\right) , \end{aligned}$$

we compute, for $X \in \partial B$,

$$\begin{aligned} {\widetilde{F}}(X)= & {} {\widetilde{F}}_2(X)+\frac{1}{\delta \varepsilon _c}\left( \frac{1}{2}I-{\mathcal {K}}^{k_c\delta ,*}_B\right) \left( {\mathcal {S}}^{k_c\delta }_B\right) ^{-1}[{\widetilde{F}}_1](X) \\= & {} -\frac{1}{\delta \varepsilon _m}\frac{\partial e^{ik_m d\cdot (z + \delta X)}}{\partial \nu _X}-\frac{1}{\delta \varepsilon _c}\left( \frac{1}{2}I-{\mathcal {K}}^*_B + {\mathcal {O}}\left( \left( k_c \delta \right) ^2 \log (k_c \delta )\right) \right) \\&\qquad \left( {\mathcal {L}}_B+{\mathcal {U}}_{k_c\delta }+ {\mathcal {O}}\left( \left( k_c \delta \right) ^2 \log (k_c \delta )\right) \right) \left[ e^{ik_m d\cdot (z + \delta X)}\right] \\= & {} -\frac{1}{\delta \varepsilon _m}\nu _X\cdot \nabla e^{ik_m d\cdot (z + \delta X)}-\frac{1}{\delta \varepsilon _c}\left( \frac{1}{2}I-{\mathcal {K}}^*_B\right) \widetilde{{\mathcal {S}}}^{-1}_B\left[ e^{ik_m d\cdot (z + \delta X)}\right] \\&+{\mathcal {O}}\left( \omega ^2 \delta c ^{-2} \log \left( \omega \delta c^{-1}\right) \right) \\= & {} - \frac{1}{\varepsilon _m}\nu _X\cdot \nabla e^{ik_m d\cdot z}-\frac{e^{ik_m d\cdot z}}{\delta \varepsilon _c} \left( \frac{1}{2}I -{\mathcal {K}}^*_B\right) \widetilde{{\mathcal {S}}}^{-1}_B\left[ \chi (\partial B)\right] \\&-\frac{\nabla e^{ik_m d\cdot z}}{\varepsilon _c}\cdot \left( \frac{1}{2}I-{\mathcal {K}}^*_B\right) \widetilde{{\mathcal {S}}}^{-1}_B[X]+{\mathcal {O}}\left( \omega ^2 \delta c ^{-2} \log \left( \omega \delta c^{-1}\right) \right) \\= & {} -\frac{1}{\varepsilon _m}\nu _X\cdot \nabla e^{ik_m d\cdot z}+\frac{1}{\varepsilon _c}\nu _X\cdot \nabla e^{ik_m d\cdot z}+{\mathcal {O}}\left( \omega ^2 \delta c ^{-2} \log \left( \omega \delta c^{-1}\right) \right) , \end{aligned}$$

where we used $\left( \frac{1}{2}I-{\mathcal {K}}^*_B\right) \widetilde{{\mathcal {S}}}^{-1}_B[\chi (\partial B)]=0$ and $\left( \frac{1}{2}I-{\mathcal {K}}^*_B\right) {\mathcal {U}}_{k \delta }=0$. It is immediate to see that $\left( \frac{1}{2}I-{\mathcal {K}}^*_B\right) \widetilde{{\mathcal {S}}}^{-1}_B[X] =-\nu _X$, indeed assuming there exists $\phi \in {\mathcal {H}}^*(\partial B)$ such that $\widetilde{{\mathcal {S}}}^{-1}_B[X]=\phi$, then $\widetilde{{\mathcal {S}}}_B[\phi ]=X$ so $\left. \partial \widetilde{{\mathcal {S}}}_B[\phi ]/\partial \nu _X \right| _-= \nu _X$ and using jump conditions for $\phi \ne \varphi _0$ we get $\left. \partial \widetilde{{\mathcal {S}}}_B[\phi ]/\partial \nu _X \right| _-=\left. \partial {\mathcal {S}}_B[\phi ]/\partial \nu _X \right| _-=\left( -\frac{1}{2}I+{\mathcal {K}}^*_B\right) [\phi ]=\nu _X$.

$$\square $$

## Proof of Theorem 5

We need the following lemma:

### Lemma 14

The Hankel function has the following asymptotics as $x\rightarrow +\infty$:

30 $$\begin{aligned} H_0^{(1)}(x)=\sqrt{\frac{2}{\pi x}}e^{i(x-\pi /4)}+{\mathcal {O}}\left( \frac{1}{x}\right) . \end{aligned}$$

For *x* large and $y \in \partial D$:

$$\begin{aligned} {\mathcal {S}}_D^{\frac{\omega }{c}}\left[ \varphi _j\right] (x)&= - \frac{i}{4} \int _{\partial D} H_0^{(1)}\left( \omega c^{-1}|x-y|\right) \varphi _j (y) \mathrm {d}\sigma (y) \\&\sim - \frac{i\sqrt{2}}{4\sqrt{\pi }} \int _{\partial D} \frac{e^{i\left( \omega c^{-1} |x-y|-\pi /4\right) }}{\sqrt{\omega c^{-1} |x-y|}}\varphi _j (y) \mathrm {d}\sigma (y). \end{aligned}$$

### Lemma 15

As $\omega \delta c^{-1} \rightarrow 0$, *F* defined in (7) admits the following asymptotic expansion:

$$\begin{aligned} F(x) = \frac{f(\omega )}{\delta } \left[ i \omega \delta c^{-1} e^{i \omega c^{-1} d\cdot z}\left( \frac{1}{\varepsilon _c}-\frac{1}{\varepsilon _m}\right) d \cdot \nu _x +{\mathcal {O}}\left( \left( \omega \delta c^{-1} \right) ^2 \log \left( \omega \delta c^{-1} \right) \right) \right] , \quad x \in \partial D. \end{aligned}$$

### Proof

See Appendix C.4. $$\square $$

### Lemma 16

As $\omega \delta c^{-1} \rightarrow 0$, the scalar field admits the following asymptotic expansion:

$$\begin{aligned} u^\text {sca}(x,\omega ) \approx \frac{e^{-i\pi /4}}{\sqrt{8\pi c}} \sum _{j=1}^J \left\langle d \cdot \nu ,\varphi _j\right\rangle _{{\mathcal {H}}^*(\partial D)} \varXi _j(x,\omega ), \end{aligned}$$

where the modes $\varXi _j$ are defined by

$$\begin{aligned} \varXi _j(x,\omega ):=\int _{\partial D} \frac{\varphi _j(y)}{\sqrt{|x-y|}} \frac{f(\omega )\sqrt{\omega }}{(\lambda (\omega )-\lambda _j(\omega \delta ))} e^{i\omega c^{-1}(|x-y|+d\cdot z)} \mathrm {d}\sigma (y), \end{aligned}$$

and $$\lambda _j(\omega \delta ) := \lambda _j - \left( \omega \delta c^{-1}\right) ^2\log {\left( \omega \delta c^{-1}\right) }\alpha _j +{\mathcal {O}}\left( \left( \omega \delta c^{-1}\right) ^2\right) $$.

### Proof

Since $\left\langle \nu ,\varphi _0\right\rangle _{{\mathcal {H}}^*(\partial D)}=0$, the zeroth term vanishes in the summation. $\square$

The goal of this section is to establish a resonance expansion for the low-frequency part of the scattered field in the time domain. Introduce, for $0<\epsilon <\rho$, the truncated inverse Fourier transform of the scattered field $u^{\text {sca}}$ given by

$$\begin{aligned} P_{\rho ,\epsilon }\left[ {\widehat{u}}^\text {sca}\right] (x,t)=\int _{-\rho }^{-\epsilon } u^{\text {sca}}(x,\omega ) e^{-i\omega t} \mathrm {d}\omega +\int _{\epsilon }^{\rho } u^{\text {sca}}(x,\omega ) e^{-i\omega t} \mathrm {d}\omega . \end{aligned}$$

Recall that *z* is the centre of the resonator and $\delta$ its radius. Let us define

$$\begin{aligned} t_0^\pm (d,x):=\frac{1}{c}\left( |x-z|+d\cdot z \pm 2\delta \right) \pm C_1, \end{aligned}$$

the time it takes to the signal to reach first the scatterer and then observation point *x*. The term $\pm 2 \delta /c$ accounts for the maximal timespan spent inside the particle.

### Proof

We have

31 $$\begin{aligned}&P_{\rho ,\epsilon }\left[ {\widehat{u}}^\text {sca}\right] (x,t) \nonumber \\&\quad \sim \sum _{j=1}^J \frac{ e^{-i \pi /4}}{\sqrt{8 \pi c}} \left\langle d \cdot \nu ,\varphi _j\right\rangle _{{\mathcal {H}}^*(\partial D)}\left[ \int _{-\rho }^{-\epsilon } \varXi _j(x,\omega )e^{-i\omega t}\mathrm {d}\omega +\int _{\epsilon }^{\rho } \varXi _j(x,\omega )e^{-i\omega t}\mathrm {d}\omega \right] .\nonumber \\ \end{aligned}$$

For $j\ge 1$, let us compute the contribution of one mode $\varXi _j(x,\omega )$. We want to apply the residue theorem to get an asymptotic expansion in the time domain. Note that:

$$\begin{aligned}&\int _{-\rho }^{-\epsilon } \varXi _j(x,\omega ) e^{-i\omega t} \mathrm {d}\omega +\int _{\epsilon }^{\rho } \varXi _j(x,\omega ) e^{-i\omega t} \mathrm {d}\omega \\&\quad = \oint _{{\mathcal {C}}^{\pm }} \varXi _j(x,\varOmega ) e^{-i\varOmega t}\mathrm {d}\varOmega -\int _{{\mathcal {C}}_\rho ^{\pm }} \varXi _j(x,\varOmega ) e^{-i\varOmega t} \mathrm {d}\varOmega -\int _{{\mathcal {C}}_\epsilon ^{\pm }} \varXi _j(x,\varOmega ) e^{-i\varOmega t} \mathrm {d}\varOmega , \end{aligned}$$

where the integration contours ${\mathcal {C}}_\rho ^{\pm }$ and ${\mathcal {C}}_\epsilon ^{\pm }$ are semi-circular arcs of radius $\rho$ and $\epsilon$, respectively, in the upper (+) or lower (-) half-planes, and ${\mathcal {C}}^{\pm }$ is the closed contour defined as ${\mathcal {C}}^{\pm }:={\mathcal {C}}_\rho ^{\pm }\cup {\mathcal {C}}_\epsilon ^{\pm }\cup [-\rho ,-\epsilon ]\cup [\epsilon ,\rho ]$. The integral on the closed contour is the main contribution to the scattered field by the mode and can be computed using the residue theorem to get, for $\rho \ge \max _{j \in \{1,\ldots ,J\}}\mathfrak {R}[\varOmega _j^\pm (\delta )]$ and $0<\epsilon \le \min _{j\in \{1,\ldots ,J\}} \mathfrak {R}[\varOmega _j^\pm (\delta )]$,

$$\begin{aligned} \oint _{{\mathcal {C}}^{+}} \varXi _j(x,\varOmega ) e^{-i\varOmega t} \mathrm {d}\varOmega&=0,\\ \oint _{{\mathcal {C}}^{-}} \varXi _j(x,\varOmega ) e^{-i\varOmega t} \mathrm {d}\varOmega&=2\pi i\text {Res}\left( \varXi _j(x,\varOmega )e^{-i\varOmega t},\varOmega _j^\pm (\delta )\right) . \end{aligned}$$

Since $\varOmega _j^\pm (\delta )$ is a simple pole of $\omega \mapsto \dfrac{1}{\lambda (\omega )-\lambda _j(\omega \delta )}$ we can write:

$$\begin{aligned} \oint _{{\mathcal {C}}^{-}} \varXi _j(x,\varOmega ) e^{-i\varOmega t} \mathrm {d}\varOmega&=2\pi i\text {Res}\left( \varXi _j(x,\varOmega ),\varOmega _j^\pm (\delta )\right) e^{-i\varOmega _j^\pm (\delta )t}. \end{aligned}$$

To compute the integrals on the semi-circle, we introduce:

$$\begin{aligned} X_j(y,\varOmega )=\frac{\varphi _j(y)}{\sqrt{|x-y|}} \frac{\sqrt{\omega }}{(\lambda (\omega )-\lambda _j)} \qquad y \in \partial D. \end{aligned}$$

Given the regularity of the input signal ${\widehat{f}} \in C_0^{\infty }([0,C_1])$, the Paley-Wiener theorem [46, p.161] ensures decay properties of its Fourier transform at infinity. For all $N\in {\mathbb {N}}^*$ there exists a positive constant $C_N$ such that for all $\varOmega \in {\mathbb {C}}$

$$\begin{aligned} |f(\varOmega )|\le C_N (1+|\varOmega |)^{-N}e^{C_1 |\mathfrak {I}{(\varOmega )}|}. \end{aligned}$$

Let $T:=(|x-y|+d\cdot z)/c$. We now rewrite the integrals on the large semi-circle

$$\begin{aligned} \int _{{\mathcal {C}}_\rho ^{\pm }} \varXi _j(x,\varOmega ) e^{-i\varOmega t} \mathrm {d}\varOmega =\int _{{\mathcal {C}}_\rho ^{\pm }}f(\varOmega ) \int _{\partial D} X_j(y,\varOmega ) e^{i \varOmega \left( T-t\right) }\mathrm {d}\sigma (y) \mathrm {d}\varOmega . \end{aligned}$$

We have that $t_0^-+C_1 \le T \le t_0^+-C_1$. Two cases arise. *Case 1:* For $0<t<t_0^-$ , i.e., when the signal emitted at *s* has not reached the observation point *x*, we choose the upper-half integration contour ${\mathcal {C}}^+$. Transforming into polar coordinates, $\varOmega =\rho e^{i\theta }$ for $\theta \in [0,\pi ]$, we get:

$$\begin{aligned} \left| e^{i \varOmega \left( T -t\right) }\right| \le e^{ -(t_0^--t+C_1)\mathfrak {I}(\varOmega )} \qquad \forall y \in \partial D, \end{aligned}$$

and

$$\begin{aligned} \left| \int _{{\mathcal {C}}_\rho ^{+}} \varXi _j(x,\varOmega ) e^{-i\varOmega t} \mathrm {d}\varOmega \right|&\le \int _0^\pi \rho \left| f\left( \rho e^{i\theta }\right) \right| e^{-\rho (t_0^--t+C_1)\sin {\theta }}\int _{\partial D}\vert X_j(y, \rho e^{i\theta }) \vert \mathrm {d}\sigma (y) \mathrm {d}\theta ,\\&\le \rho C_N(1+\rho )^{-N} \delta \max _{\theta \in [0,\pi ]}{\left| X_j\left( \cdot , \rho e^{i\theta }\right) \right| _{L^{\infty }(\partial D)}} \pi \frac{1-e^{-\rho (t^-_0-t)}}{\rho (t^-_0-t)}, \end{aligned}$$

where we used that for $\theta \in [0,\pi /2]$, we have $\sin {\theta } \ge 2\theta /\pi \ge 0$ and $-\cos {\theta }\le -1+2\theta /\pi$. The usual way to go forward from here is to take the limit $\rho \rightarrow \infty$, and get that the limit of the integral on the semi-circle is zero. As in the three-dimensional case, we work in the quasi–static approximation here, and our modal expansion is not uniformly valid for all frequencies. So we have to work with a fixed maximum frequency $\rho$. However, the maximum frequency $\rho$ depends on the size of the particle via the hypothesis $\rho \le c\delta ^{-1}$. Since *N* can be taken arbitrarily large and that $X_j$ behaves like a polynomial in $\rho$ *whose degree does not depend on j*, we get that, uniformly for $j\in [1,J]$:

$$\begin{aligned} \left| \int _{{\mathcal {C}}_\rho ^{+}} \varXi _j(x,\varOmega ) e^{-i\varOmega t} \mathrm {d}\varOmega \right| = {\mathcal {O}}\left( \frac{\delta }{t_0^--t} \rho ^{-N}\right) . \end{aligned}$$

For the upper-half semi-circle of radius $\epsilon$, we also transform into polar coordinates with the change of variable $\varOmega =\epsilon e^{i\theta }$, for $\theta \in [0,\pi ]$, and get:

$$\begin{aligned} \left| \int _{{\mathcal {C}}_\epsilon ^{+}} \varXi _j(x,\varOmega ) e^{-i\varOmega t} \mathrm {d}\varOmega \right| \le \epsilon C_N(1+\rho )^{-N} \delta \max _{\theta \in [0,\pi ]}{\left| X_j\left( \cdot , \epsilon e^{i\theta }\right) \right| _{L^{\infty }(\partial D)}} \pi \frac{1-e^{-\epsilon [(t^-_0-t)]}}{\epsilon (t^-_0-t)}. \end{aligned}$$

*Case 2:* For $t>t_0^+$ , we choose the lower-half integration contour ${\mathcal {C}}^-$. Transforming into polar coordinates, $\varOmega =\rho e^{i\theta }$ for $\theta \in [\pi ,2\pi ]$, we get

$$\begin{aligned} \left| e^{i \varOmega \left( T -t\right) }\right| \le e^{ (t-t_0^+-C_1) \mathfrak {I}(\varOmega )} \qquad \forall y \in \partial D^2, \end{aligned}$$

and

$$\begin{aligned} \left| \int _{{\mathcal {C}}_\rho ^{-}} \varXi _j(x,\varOmega ) e^{-i\varOmega t} \mathrm {d}\varOmega \right|&\le \int _\pi ^{2\pi }\rho \left| f\left( \rho e^{i\theta }\right) \right| e^{\rho (t-t_0^+)\sin {\theta }}\int _{\partial D}\vert X_j(y, \rho e^{i\theta }) \vert \mathrm {d}\sigma (y) \mathrm {d}\theta ,\\&\le \rho C_N(1+\rho )^{-N}\delta \max _{\theta \in [\pi ,2\pi ]}{\left| X_j\left( \cdot , \rho e^{i\theta }\right) \right| _{L^{\infty }( \partial D)}}\pi \frac{1-e^{-\rho (t-t_0^+)}}{\rho (t-t_0^+)}. \end{aligned}$$

Exactly as in Case 1, we cannot take the limit $\rho \rightarrow \infty$. However, the maximum frequency $\rho$ depends on the size of the particle via the hypothesis $\rho \le c\delta ^{-1}$. Using the fact that *N* can be taken arbitrarily large and that $X_j$ behaves like a polynomial in $\rho$ *whose degree does not depend on j*, we get that, uniformly for $j\in [1,J]$:

$$\begin{aligned} \left| \int _{{\mathcal {C}}_\rho ^{-}} \varXi _j(x,\varOmega ) e^{-i\varOmega t} \mathrm {d}\varOmega \right| = {\mathcal {O}}\left( \frac{\delta }{t} \rho ^{-N}\right) . \end{aligned}$$

For the lower-half semi-circle of radius $\epsilon$, we also transform into polar coordinates with the change of variable $\varOmega =\epsilon e^{i\theta }$, for $\theta \in [0,\pi ]$, and get:

$$\begin{aligned} \left| \int _{{\mathcal {C}}_\epsilon ^{-}} \varXi _j(x,\varOmega ) e^{-i\varOmega t} \mathrm {d}\varOmega \right| \le \epsilon C_N(1+\rho )^{-N} \delta \max _{\theta \in [\pi ,2\pi ]}{\left| X_j\left( \cdot , \epsilon e^{i\theta }\right) \right| _{L^{\infty }(\partial D)}} \pi \frac{1-e^{-\epsilon (t-t^+_0)}}{\epsilon (t-t^+_0)}. \end{aligned}$$

The result of Theorem 5 is obtained by summing the contribution of all the modes. $\square$

## Declarations

### Data availability

The data supporting the findings of this study were generated through matlab and are available from the corresponding author on request.
